# Syenthis, structural, magnetic, optical, and dielectric properties of ag and cr substituted Co-ferrite nanoparticles

**DOI:** 10.1038/s41598-025-22606-x

**Published:** 2025-11-04

**Authors:** R. El-Shater, N. I. A. Sad, E. K. Abdel-khalek, T. M. Meaz, M. K. El-Nimr, F. Fakhry

**Affiliations:** 1 Physics Department, Faculty of Science, Tanta University, Tanta, 31527 Egypt; 2 Physics Department, Faculty of Science, Al-Azhar University, Nasr City, 11884 Cairo Egypt

**Keywords:** Cr-Ag-Co nano-ferrite particles, Optical properties, Magnetic properties, Dielectric properties, Materials science, Nanoscience and technology, Physics

## Abstract

The co-precipitation method was used to synthesize **Co**_**1−2x**_**Ag**_**x**_**Cr**_**x**_**Fe**_**2**_**O**_**4**_, Co-ferrite doped with Ag and Cr (where x = 0.0, 0.02, 0.04, 0.06, 0.08, and 1). The obtained samples’ single phase of the cubic spinel structure was confirmed by XRD analysis. The crystallites in the samples ranged in size from 11 to 18 nm. XPS analysis of the prepared samples verified the existence of many equivalents of metallic ions. TEM images displayed the nanocrystalline nature of the prepared samples and gave an estimation of their size and shape. The vibrational stretching of tetrahedral and octahedral complexes is the cause of two absorption bands that are revealed by FTIR spectroscopy even after Ag and Cr replacement. The magnetic properties of the VSM data indicate that the saturation magnetization decreases as the Cr and Ag levels rise. All of the produced samples have low band gap energy values according to UV-vis absorbance spectroscopy, which makes them attractive candidates for catalytic applications. Between 10 Hz and 10 MHz, the produced nanoparticles show dielectric characteristics. As the frequency increases, the dielectric readings decrease, but the ac conductivity shows the opposite pattern. As the frequency goes up, the Tanδ values go down a little until they reach very low levels, while the ε′ values get closer to a steady value. This implies the possibility of creating radio frequency-dependent electronics using a low-loss and low-permittivity dielectric substrate.

## Introduction

 These days, a variety of fields from the biomedical to the industrial are interested in spinel ferrite nanomaterials due to their remarkable structure and electrical and magnetic properties. Several factors that influence these characteristics are chemical composition, preparation conditions, magnetic ion electronic structure, lattice crystal structure, sintering temperature, and time^1^. Due to its ferromagnetic properties, ferrite is widely employed as a semiconductor in the radio (30 kHz to 300 GHz) and micro (300 MHz to 300 GHz) frequency ranges. The spinel crystal structure of ferrites is formed by the combination of a divalent metallic cation with a trivalent (Fe^3+^) cation. The formula for spinel ferrites is MFe_2_O4, where M stands for Fe^2+^, Co^2+^, etc. In spinel ferrites, the two-site crystal structure is composed of the divalent and trivalent transition metal ions that occupy the octahedral site (B) and the tetrahedral site (A).

The substitution of transition metal ions at sites A and B has a substantial effect on the structural, magnetic, chemical, and physical properties of ferrites^2^. One of the most interesting ferrites, according to a survey of the literature, is CoFe_2_O_4_, whose degree of inversion depends on heat treatment^3^. Cobalt ferrites are hard magnetic materials with an inverse spinel ferrite that have great physical and chemical stabilities, strong coercivity, and saturation magnetism^4^. CoFe_2_O_4_ has high magneto-crystallite anisotropy, which is one of the main characteristics that suit it for biological applications^5,6^. CoFe_2_O_4_’s enhanced electrical and magnetic characteristics make it a viable material for high-density recording^7^. The electric and magnetic characteristics of a material can be changed by just a small quantity of impurity^8^. Consequently, the magnetic and electrical characteristics of nano CoFe_2_O_4_ are affected by the substitution process between the transition metal and the rare-earth ions^9–17^.

The main reason for the observed modification of the CoFe_2_O_4_ magnetic characteristics after Cr^3+^ ions are added is that these ions (ionic radius 0.63 A ˙) always occupy the Fe^3+^ (ionic radius 0.67 A) (B) site and control the parameters of magnetic et al. Toksha^18^. The physical characteristics of spinel cobalt ferrite were modified when cobalt cations were partially substituted with chromium and silver, which modified the cation distribution in the tetrahedral (A) and octahedral (B) sites^19^. The thermoelectric and electrical properties of Cr-substituted CoFe_2_O_4_nanoparticles produced by the co-precipitation technique were examined by Anis-ur-Rehman et al^20^. and Pervaiz et al^21^..

In this study, the Co-precipitation approach was used to create pure and Cr, Ag substitution CoFe_2_O_4_ nanoparticle samples for the system **Co**_**(1−x)**_**Ag**_**x**_**Cr**_**x**_**Fe**_**2**_**O**_**4**_ for varying Cr and Ag concentrations (x = 0.0, 0.02, 0.04, 0.06,0.08, or 0.1). It is necessary to comprehend the relationship between structure, particle size, dielectric qualities, and magnetic properties to design new magnetic materials. The current study aimed to clarify the effects of Ag^+^ and Cr^3+^ ion concentration on the structural, dielectric, and magnetic properties of Co-ferrite nanoparticles produced by the co-precipitation method.

## Materials and methods

The **Co**_**1−2x**_**Ag**_**x**_**Cr**_**x**_**Fe**_**2**_**O**_**4**_ was synthesized using the co-precipitation method, which is one of the best methods due to the high ultrafine samples obtained at low sintering temperature and the straightforward experimental arrangement. The concentrations of Ag and Cr were x = 0.0, 0.02, 0.04, 0.06, 0.08, and 0.1. The NaOH solution was added dropwise as a precipitant after dissolving ferric nitrate nonahydrate, cobalt nitrate hexahydrate, silver nitrate, and chromium nitrate hexahydrate aqueous solutions in distilled water. Using a magnetic stirrer, the stirring speed was kept constant at 200 rpm throughout the reaction, and all of the metal nitrate solutions were combined and heated to 85 °C. Drops of NaOH solution were added to the nitrate solutions for the reaction, so the pH of the solution was maintained at ~ 10.5 during precipitation to ensure that precipitation occurred. The mixture was agitated at a steady pace and heated to 80 °C for three hours. To remove the unreacted residual salts, the precipitated ferrite was gathered and repeatedly cleaned with distilled water. The precipitate nanoparticles were dried for 24 h at 60 °C.

In the Central Laboratory at Tanta University in Egypt, The **Co**_**1−2x**_**Ag**_**x**_**Cr**_**x**_**Fe**_**2**_**O**_**4**_ nanoparticles with (x = 0.0, 0.02, 0.04, 0.06, 0.08, and 0.1) were identified structurally and their phase was analyzed using an X-ray diffractometer (Rigaku, Ultima IV X-ray diffractometer) (XRD) with Cu-Kα radiation at 30 mA and 40 kV (λ = 1.540598 Å) in the range of 2θ = 20˚ to 80˚ was investigated with a step of 0.02° for 4s. At the Nano Center in Egypt, the chemical composition of the samples was recorded using X-ray photoelectron spectroscopy (Ulvac-Phi QuanteraSXM) (XPS) with AlKα. Using a transmission electron microscope (JEOL-TEM-2100 F microscope with 200 kV), the morphology, particle size, and area distribution were determined using a C-C peak at 284.8 eV for charge correction, which yields the chemical binding states. The Central Laboratory of Tanta University in Egypt used Fourier transform infrared spectroscopy (FT-IR) (Bruker FT-IR TENSOR 27) was employed to examine the chemical bonding of the samples, functional groups, and material identification, where the spectra range from 200 to 2000 cm^−1^. At the National Research Center in Egypt, a Diffuse Reflectance Spectrophotometric Study (DRS), a type of UV spectrophotometer, was created to assess the linear optical features and estimate the band gap energies. A Vibrating Sample Magnetometer (VSM) was used to measure the magnetic properties at room temperature. Using the Jasco model V-570 scanning spectrometer, DRS was performed. The ferrite powders were pressed in an appropriate die to measure the dielectric properties at different frequencies using Alpha Analyzer, Novocontrol GmbH, at the National Research Center, Egypt. The dielectric constant, dielectric loss factor tan(δ), and ac conductivity $\:{\sigma\:}_{ac}$’ and impedance measurements (real$\:{Z}^{{\prime\:}}$ and imaginary $\:{Z}^{"}$ Impedance) were evaluated at room temperature.

## Results and discussion

### X-ray diffraction (XRD) studies

**Fig. 1:**
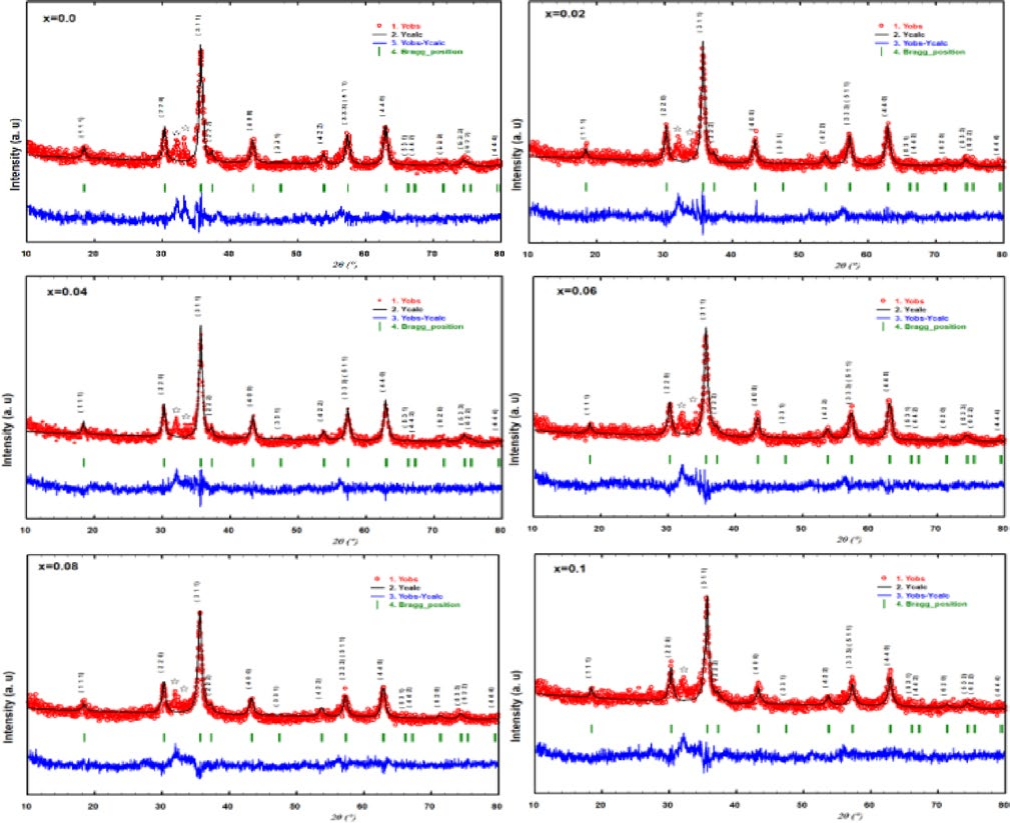
XRD patterns with Rietveld Refinement of the as-prepared spinel ferrite **Co**_**1−2x**_**Ag**_**x**_**Cr**_**x**_**Fe**_**2**_**O**_**4**_.

The XRD spectra with Rietveld Refinement displayed in Fig. 1 reveal that all of the samples are cubic mixed spinel ferrite. Sharp peaks identified as (220), (311), (400), (511), and (440) planes are also visible in the spectra; they are in good agreement with the JCPDS card (22–1086)^22^.

 Equation (1) was used to get the lattice parameter (*a*) from the peak (311) reflection by (*d*) spacing, which is shown in Table 1^23^.

1 $$\:a=d\sqrt{{h}^{2}+{k}^{2}+{l}^{2}}$$

where the interplanar distance, (*d*), can be determined using Bragg’s rule (2), and the Miller indices are (hkl).

2 $$\:2{d}_{hkl}sin\theta\:=n\lambda\:$$

The Bragg angles are denoted by (θ). Williamson-Hall analysis (W–H) is used to separate the crystallite size and strain-induced deformation peak by taking the peak width broadening as a function of 2θ into account, The total broadening (β) of the peak is the result of the combined broadening due to crystallites size (β_R_) and broadening due to lattice strain (β_ε_). As a result, total broadening is obtained by^24^:

3 $$\:\beta\:={\beta\:}_{R}+{\beta\:}_{\epsilon\:}=\frac{k\lambda\:}{Rcos\theta\:}+\frac{4\epsilon sin\theta\:}{cos\theta\:}$$

**Fig. 2:**
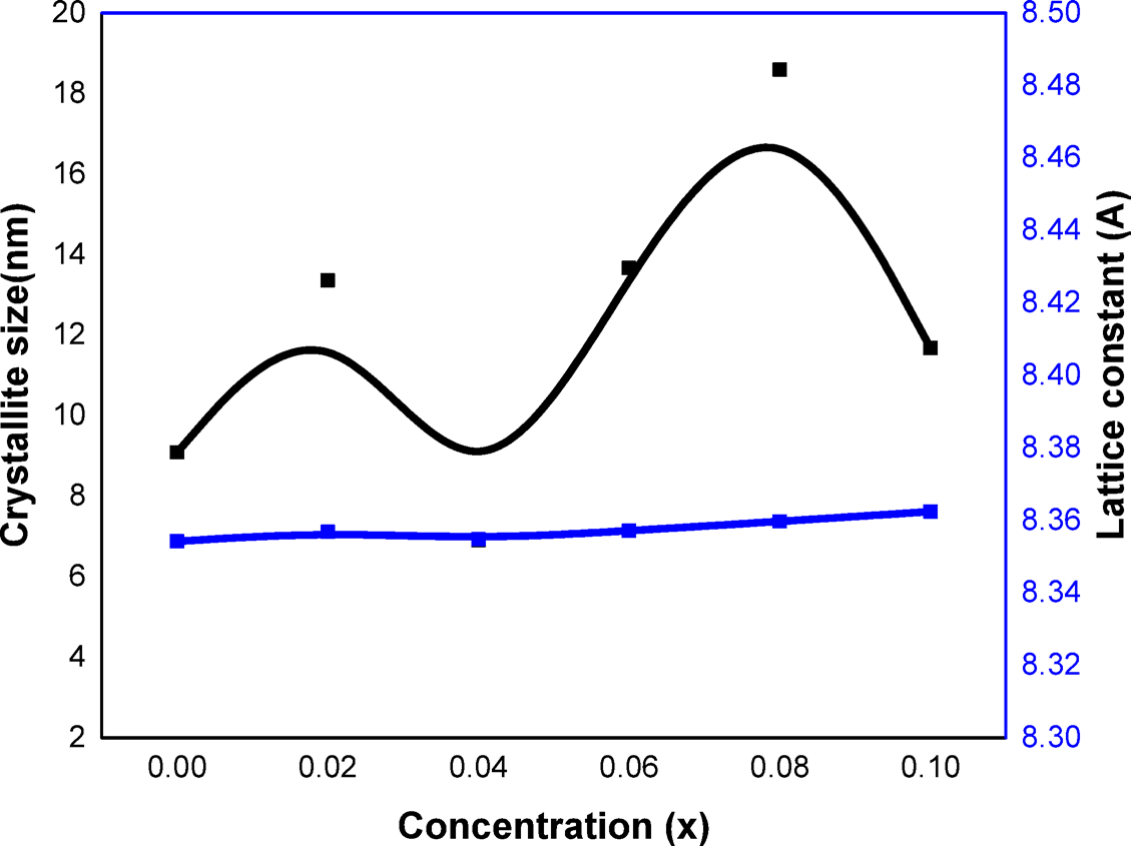
Variation of crystallite size (R) and lattice constant (*a*) with concentration (x).

As illustrated in Fig. 2, The lattice parameter (a) of pure CoFe_2_O_4_ (x = 0.0) is marginally smaller than the lattice parameter of Ag, Cr doped CoFe_2_O_4_, except for sample x = 0.04’s lattice parameter which is smaller than pure CoFe_2_O_4_ and results consistently in a decrease in lattice constant upon substitution by Cr^3+^ ions^23,25^. Additional nonlinearity of the lattice constant with the concentration of Cr^3+^ ions for non-inverse or non-fully normal systems has been reported in the literature^26^. The distribution of cations may have changed as a result of Ag and Cr being substituted in the spinel structure, which could explain this.

The crystallite size (R) of Ag and Cr-doped CoFe_2_O_4_ starts at 13.095 nm and gradually falls, except for (x = 0.08 and 0.1) where it increases, as illustrated in Fig. 2. In Ag and Cr-doped CoFe_2_O_4_, larger Ag nanoparticles might serve as resistive centers at the grain boundaries, preventing the surface energy needed for crystallite formation from being released. Additionally, the variation in crystallite size with increasing Ag and Cr concentrations may be caused by strain and lattice disorder in the produced samples; this strain variation may be influenced by the behavior of lattice constants and the ion jump length (Table 2)^27^. The real proportion of silver that contributed to the composition may determine the variance in X-ray properties^28^.

The following Eqs. (4,5,6,7,8) were used to calculate the structural parameters using XRD data: X-ray density (D_XRD_), experimental density (D_exp_), porosity (P), experimental surface area (S_XRD_), and lattice strain (ε).

4 $$\:{D}_{XRD}=\frac{ZM}{{N}_{A}{a}_{exp}^{3}}$$

5 $$\:{D}_{exp}=\frac{m}{\left(\pi\:{r}^{2}h\right)}$$

6 $$\:P=1-\frac{{D}_{exp}}{{D}_{XRD}}$$

7 $$\:{S}_{XRD}=\frac{6000}{R{D}_{XRD}}$$

8 $$\:\epsilon\:=\frac{\beta\:cos\theta\:}{4sin\theta\:}$$

The atoms per unit cell are denoted by (Z), Avogadro’s number by (N), the composition’s molecular weight by (M), the mass by (m), the radius by (r), the thickness of the sample’s pellet by (h), the diffraction angle by (θ), and the full-width at half-maximum (FWHM) of the XRD peak by (β) (311). A record of the calculated values can be found in Table 1.

**Fig. 3:**
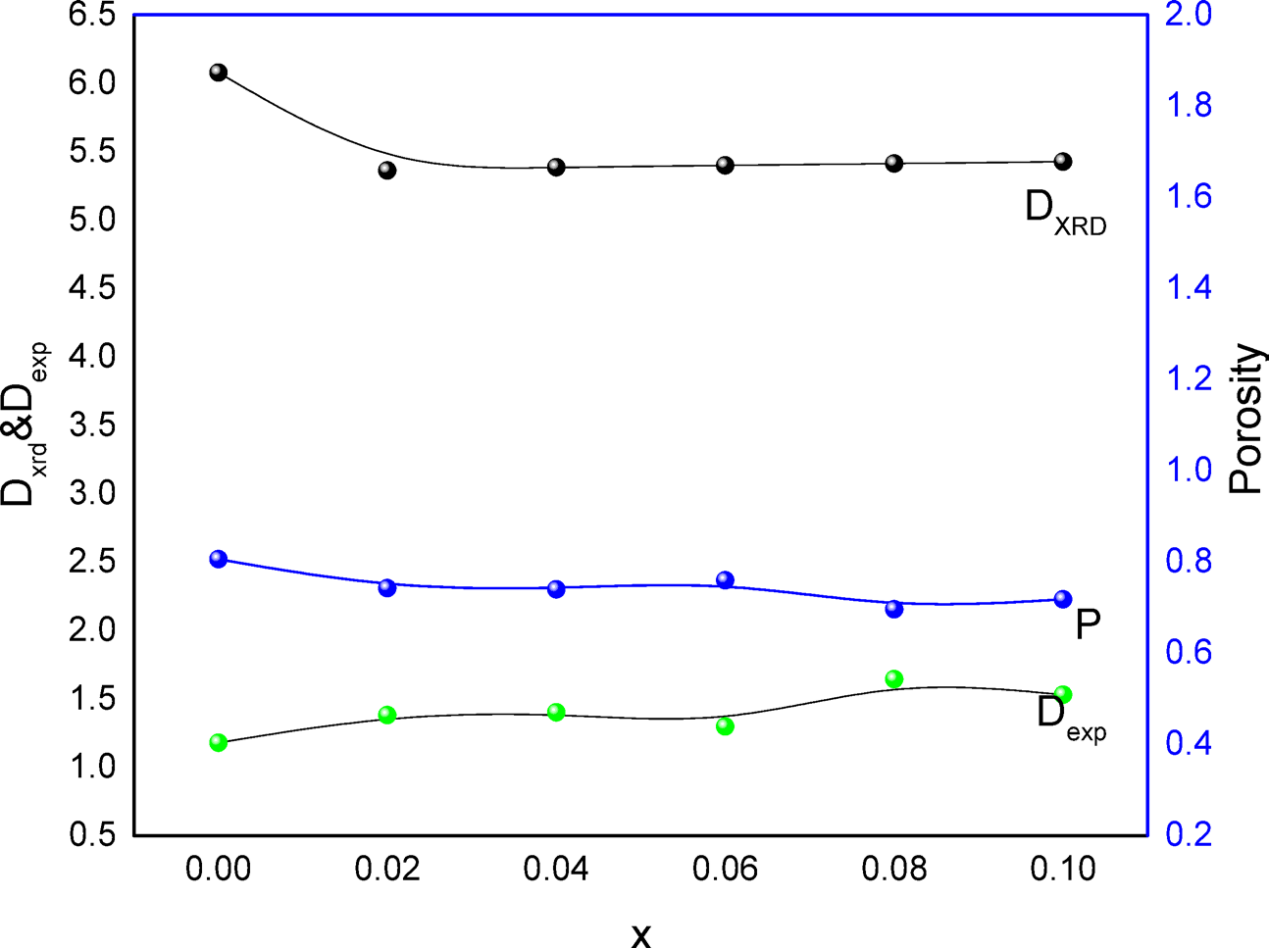
Variation of X-ray density (D_XRD_), Experimental density D_x,_ and porosity (P) with concentration(x).

According to the samples’ lattice constant and molecular weight, there is a modest increase in x-ray density (D_XRD_) due to iron and silver substitution, as illustrated in Fig. 3. The substitution of low atomic weight Cr^3+^ions (51.996) and high atomic weight Ag^+^ ions (1078.868) for Co^2+^ions (58.933) will therefore raise density from 5.14 to 5.20 g/cm^3^. This is because some pores that formed during the preparation process are the reason why the experimental density (D_EXP_) values are lower than the x-ray density (D_XRD_) values, which are consistent with previous findings^26^. Given that (D_XRD_) behaves inversely with lattice constant^26^, the high porosity values seen in Table 1 are caused by the significant discrepancy between (D_XRD_) and (D_exp_).

**Fig. 4:**
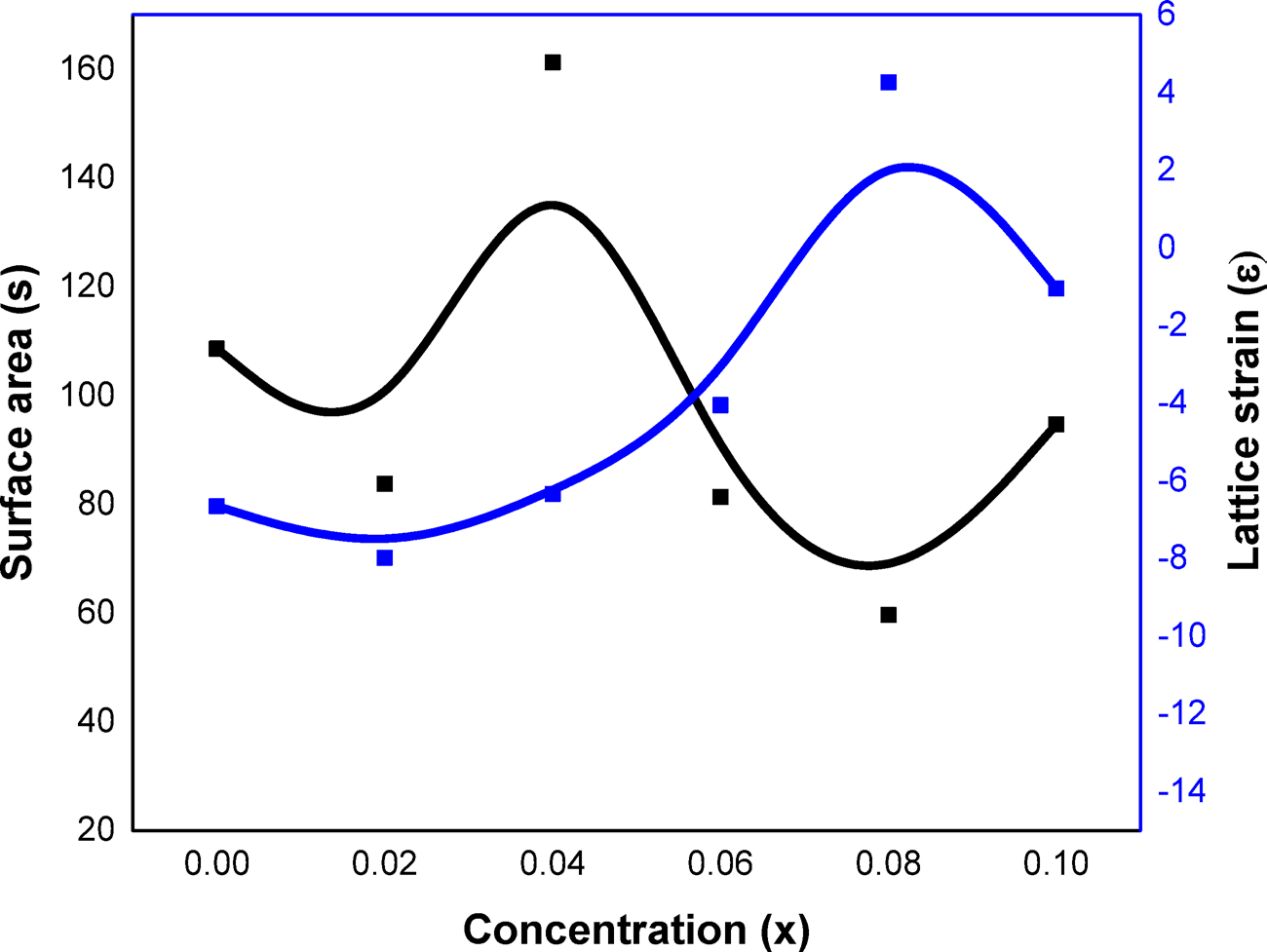
Variation of specific surface area (S) and lattice strain (ε) with concentration(x).

As illustrated in Fig. 4, the behavior of specific surface area (S). Swelling until x = 0.04 and then declining, this behavior is consistent with that of crystalline size. As specific surface area (S) and the lattice strain (ε) reduced trend with x, this behavior is dependent on the crystalline size (R) inversely.

The degree of crystallinity ($\:{X}_{c}$) of all manufactured samples is presented in Table 1 and was calculated by integrating the areas under crystalline (Ac) and amorphous (Aa) from the XRD pattern and using the Eq. (9)^29^:

9 $$\:{X}_{c}=\frac{{A}_{c}}{{(A}_{c}+{A}_{a})}$$

The decrese of crystallinity may be according to the increasing Cr and Ag content.

According to the location of the cations in the crystal lattice, spinels can have different physical and chemical properties^30^. Valence bond balance, Ion radius, ordering, electron layer structure, and other properties are frequently related to metal cation distribution rules.

The cationic distribution of the generated material according to Rietveld Refinement and its favored sites are shown in Table 2. The metallic ions Fe^2+^, Co^3+^, Ag^+^, and Cr^3+^ all favor occupying the octahedral site in the system Co_(1−x)_Ag_x_Cr_x_Fe_2_O_4_, while Co^+2^ and Fe^+3^ ions occupy the tetrahedral site.

To verify the validity of the estimated cation distribution of the tetrahedral and octahedral sites, the theoretical lattice parameter “a_th_” and the site mean ionic radii (r_A_ and r_B_) were calculated by using the following Eqs. (10, 11, 12):

10 $$\:{r}_{A}=\left[{f}_{C}\left({Fe}^{3+}\right).r\left({Fe}^{3+}\right)\right]+\left[{f}_{C}\left({Co}^{2+}\right).r\left({Co}^{2+}\right)\right]$$

11 $$\begin{aligned}\:{r}_{B}&=0.5\{\left[{f}_{C}\left({Fe}^{3+}\right).r\left({Fe}^{3+}\right)\right]+\left[{f}_{C}\left({Fe}^{2+}\right).r\left({Fe}^{2+}\right)\right]+\left[{f}_{C}\left({Co}^{3+}\right).r\left({Co}^{3+}\right)\right]\\&+\left[{f}_{C}\left({Cr}^{3+}\right).r\left({Cr}^{3+}\right)\right]+\left[{f}_{C}\left({Ag}^{+}\right).r\left({Ag}^{+}\right)\right]\}\end{aligned}$$

where f_C_ represents the fractional concentration and r represents the ionic radius of cations. In the tetrahedral site, the Fe^2+^ (0.63) and Fe^3+^ (0.49) have spin-up ionic radius. In the octahedral site, the Fe^2+^ (0.61), Fe^3+^ (0.55), Co^2+^ (0.65), Co^3+^ (0.54), Cr^3+^ (0.615), and Ag^+^ (1.15) have spin-down ionic radius (as shown in Table 2), and *R*_*0*_ is the anion oxygen radius^31^.

**Table 1:** The values of crystallite size(R), lattice parameter (a), unit cell volume, x-ray density (D_XRD_), experimental density (D_exp_), porosity (P), crystallite size, degree of crystallinity (X_C_), and lattice strain (ε).

X	a_th_ (Å)	a_exp_ (Å)	V (Å)	D_XRd_ (cm^3^/g)	D_exp_ (cm^3^/g)	*P*	S_XRD_	*R*_XRD_ (nm)	X_C_	ε x10^−3^
0.0	8.668	8.3542	583.06	6.077	1.178	0.806	108.61	9.09	20.86	−6.65
0.02	8.72	8.3568	583.61	5.362	1.38	0.743	83.76	13.36	20.85	−7.98
0.04	8.735	8.3548	583.19	5.385	1.40	0.740	161.3	6.91	21.22	−6.34
0.06	8.756	8.3571	583.67	5.399	1.297	0.760	81.34	13.66	19.61	−4.05
0.08	8.81	8.3597	584.21	5.413	1.644	0.696	59.62	18.59	18.14	4.26
0.1	8.832	8.3624	584.78	5.427	1.529	0.718	94.67	11.68	18.24	−1.05

**Table 2:** Cation distribution of Co_(1−x)_Ag_x_Cr_x_Fe_2_O_4_.

X	Cation distribution	*r*_A_(Å)	*r*_B_(Å)
0.0	(Co^2+^ _0.25_ Fe^3+^ _0.75_)_A_ [Co^3+^ _0.75_ Fe^2+^ _0.37_Fe^3+^_0.88_]_B_ O_4_	0.67	0.657
0.02	(Co^2+^ _0.25_ Fe^3+^ _0.75_)_A_ [Cr^3+^ _0.02_ Ag^+^ _0.01_Co^3+^_0.72_ Fe^2+^ _0.62_Fe^3+^_0.63_]_B_ O_4_	0.67	0.677
0.04	(Co^2+^ _0.25_ Fe^3+^ _0.75_)_A_ [Cr^3+^ _0.04_ Ag^+^ _0.03_Co^3+^_0.68_ Fe^2+^ _0.62_Fe^3+^_0.63_]_B_ O_4_	0.67	0.682
0.06	(Co^2+^ _0.25_ Fe^3+^ _0.75_)_A_ [Cr^3+^ _0.07_ Ag^+^ _0.06_Co^3+^_0.62_ Fe^2+^ _0.62_Fe^3+^_0.63_]_B_ O_4_	0.67	0.69
0.08	(Co^2+^ _0.25_ Fe^3+^ _0.75_)_A_ [Cr^3+^ _0.08_ Ag^+^ _0.07_Co^3+^_0.60_ Fe^2+^ _0.88_Fe^3+^_0.37_]_B_ O_4_	0.67	0.71
0.1	(Co^2+^ _0.25_ Fe^3+^ _0.75_)_A_ [Cr^3+^ _0.10_ Ag^+^ _0.10_Co^3+^_0.55_ Fe^2+^ _088_Fe^3+^_0.37_]_B_ O_4_	0.67	0.719

### X-ray photoelectron spectroscopy (XPS) studies

**Fig. 5:**
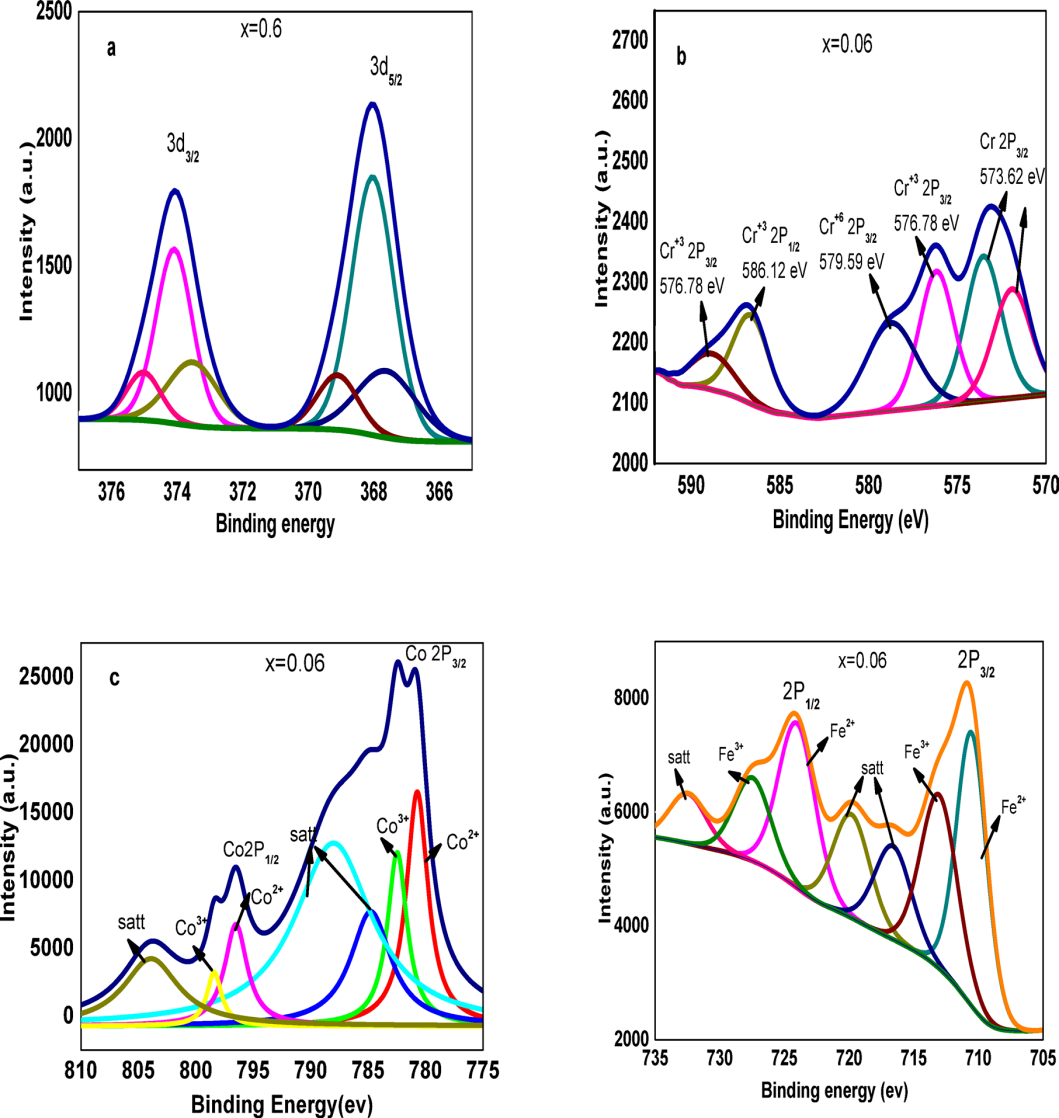
For x = 0.06 (**a**) High-resolution XPS data of an Ag 3 d, (**b**) XPS data of a Cr 2P, (**c**) XPS data of a Co 2P, and (**d**) XPS data of a Fe 2P.

The XPS spectra of as-prepared **Co**_**1−2x**_**Ag**_**x**_**Cr**_**x**_**Fe**_**2**_**O**_**4**_ spinel ferrite are displayed in Fig. 5, as the main peaks of each metal are listed in Table 2. The high-resolution XPS spectra of Ag 3 d shown in Fig. 5(a) reveal two well-separated peaks at 368.eV and 374.1 eV, which are due to 3d_5/2_ and 3d_3/2_ orbitals, respectively. These results are in agreement with the reported^32–34^.

As listed in Table 3, many Ag species are strongly supported by the many peaks of the deconvoluted Ag 3d_5/2_ spectra, the binding energy for Ag^0^ ~ 368.03, ~ 368.17, ~ 368.14, and ~ 367.03, the binding energy for AgO ~ 366.33, 368.43,~367.3, and ~ 367.61 eV, the binding energy for Ag_2_O ~ 368.63 and ~ 367.9 eV, and the binding energy for mix Ag(III) sat ~ 369.14 and ~ 369.28 eV^35,36^.

The Cr 2p spectra shown in Fig. 5(b) reveal two main peaks of the Cr 2p_3/2_ and Cr 2p_1/2_ orbitals of the two species. As listed in Table 2, the binding energy for Cr(III) ~ 576.9ev and ~ 586.8ev, while the binding energy for Cr(VI) ~ 579.8 eV and ~ 589eV^37^.

The Co 2p spectra shown in Fig. 5(c) reveal two main peaks 2p_3/2_ at 780.23 eV and 2p_1/2_ at 795.95 eV, with the spin-orbital splitting of ~ 15 eV^38^. Two satellite peaks that show a fingerprint of Co(III) cations are located at 790.08 eV and 804eV^39^. The fitting XPS curve indicates the existence of Co (IV) at 784.08 eV and 797.18 eV.

The Fe 2p spectra shown in Fig. 5(d) reveal two main peaks of Fe2p_3/2_ at 711.1 eV and Fe2p_1/2_ at 725 eV. The study of XPS samples suggests that these samples contain Fe as Fe(II) and Fe(III). Two characteristic peaks for Fe2p3/2 and Fe2p1/2 can be observed in the Fe2p spectra, with binding energies of 711.2 eV and 724.5 eV, respectively. The binding energy of Fe(II) at 2p_3/2_ and 2p_1/2_ was 711 eV and 723.8 eV, respectively, and satellite 720 eV^40^, and the binding energy of Fe(III) at 2p_3/2_ and 2p_1/2_ was 712.4 eV and 725 eV, respectively, and satellite at 732.67 eV and 717.5eV^41,42^.

**Table 3:** X-ray photoelectron spectroscopy (XPS) for ag 3 d, Fe 2p, Co 2p, and cr 2p.

Sample	Element	Equivalence	Peak position	Area
0	**Fe**	+ 2	710.65–724.08.65.08	18397.9-11535.4.9.4
		+ 3	713.37–727.36.37.36	12099.1–5375
	**Co**	+ 2	780.23–795.95.23.95	11471.1-4781.95.1.95
		+ 3	783.12–801.4	7119.2-116.2.2.2
0.02	**Fe**	+ 2	710.96–724.18.96.18	19186.1–114486.5.1.5
		+ 3	713.8–727.76.8.76	11163.9-5451.6.9.6
	**Co**	+ 2	780.6–796.18.6.18	11772.6-4822.6.6.6
		+ 3	783.12–801.51.12.51	6709.2-1662.6.2.6
	**Ag**	(Ag^0^)	368.17	308.01
		AgO	366.3- 368.43	48.7–719.2.7.2
	**Cr**	(Cr^0^)	571.47–573.62.47.62	196.91–478.1
		+ 3	576.78–586.12.78.12	595.18-391.45.18.45
		+ 6	579.59–588.98.59.98	274.95–346.4
0.04	**Fe**	+ 2	710.6–724.18.6.18	15033.2-10557.3.2.3
		+ 3	713.22–727.6	10765.2-4707.7.2.7
	**Co**	+ 2	780.31–795.9	9271.3-4163.1.3.1
		+ 3	783.3–800.39.3.39	5960.3-1012.2.3.2
	**Ag**	(Ag^0^)	368.03	1017.6
		Ag_2_O	368.63	558.5
		AgO	367.3	332.2
	**Cr**	(Cr^0^)	571.87–573.34.87.34	308.6–629.2.6.2
		+ 3	576.45–586.2	641.3-274.3.3.3
		+ 6	579.81–588.88.81.88	298.7-182.4.7.4
0.06	**Fe**	+ 2	710.52–724.05.52.05	14427.9-10546.7.9.7
		+ 3	713.04–727.46.04.46	11127.04-5330.1.04.1
	**Co**	+ 2	780.71–796.51.71.51	9571.3-5163.1.3.1
		+ 3	782.5–798.44.5.44	6260.3-1112.2.3.2
	**Ag**	(Ag^0^)	368.14	1785.9
		AgO	367.61	602.2
		Mix Ag(III) satt	369.71	119.2
	**Cr**	(Cr^0^)	571.91–573.52.91.52	495.76–627.5
		Cr^+3^	576.17–586.64.17.64	559.9-386.1.9.1
		Cr^+6^	578.73–588.81.73.81	529.3-186.8.3.8
0.08	**Fe**	+ 2	710.59–724.12.59.12	16154.02-11739.7.02.7
		+ 3	713.11–727.43.11.43	13015.8-5963.6.8.6
	**Co**	+ 2	780.39–795.97.39.97	10774.2-4595.8.2.8
		+ 3	783.4–800.99.4.99	6593.9-1224.2.9.2
	**Ag**	(Ag^0^)	368.03	2506.3
		Ag_2_O	367.9	619.9
		Mix Ag(III) satt	369.14	304.1
	**Cr**	(Cr^0^)	569.68–572.99.68.99	184.1–1704.3.1.3
		Cr^+3^	576.6–586.56.6.56	1224.2–694
		Cr^+6^	579.55–589.4	503.9-290.7.9.7
0.1	**Fe**	+ 2	711.03–724.47.03.47	17784.6-10932.1.6.1
		+ 3	713.86–727.87.86.87	10016.1-5318.5.1.5
	**Co**	+ 2	780.75–796.1	10410.04-4205.5.04.5
		+ 3	783.81–799.79.81.79	5820.5-1141.5.5.5
	**Ag**	(Ag^0^)	367.03	337.6
		Ag(I) in AgO	368.27	2813.9
		Mix Ag(III) sat	369.28	803.2
	**Cr**	(Cr^0^)	571.12–573.12.12.12	134.6–1307.7.6.7
		+ 3	576.24–587.09.24.09	967.8-593.6.8.6
		+ 6	578.56–589.58.56.58	589.26-163.47.26.47

### Electron microscope (TEM) images

**Fig. 6:**
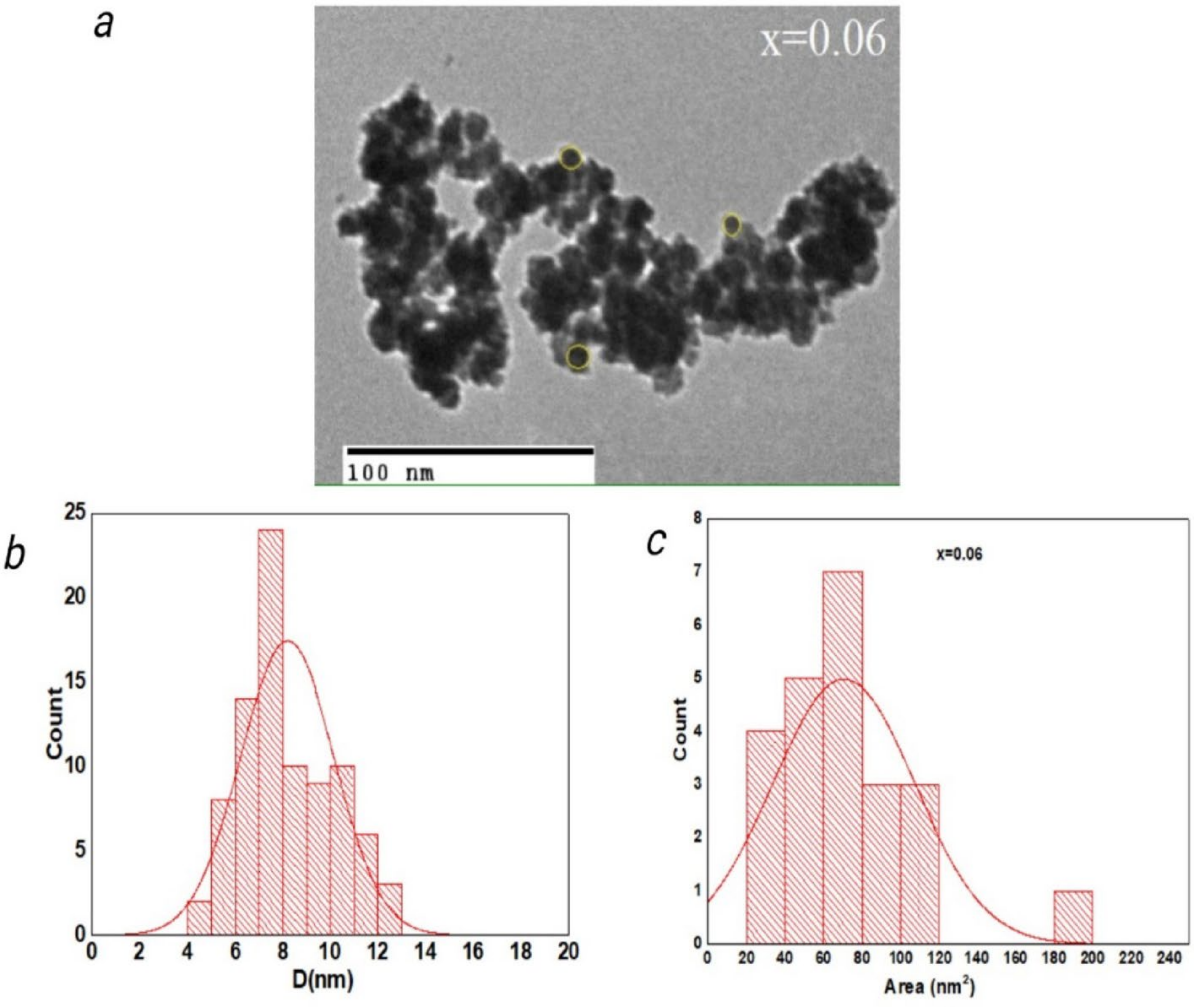
(**a**) TEM micrograph (**b**) particle size distribution (**c**) Area distribution for sample (x = 0.06).

The TEM images of the as-prepared **Co**_**1−2x**_**Ag**_**x**_**Cr**_**x**_**Fe**_**2**_**O**_**4**_ with (x = 0.06) are displayed in Fig. 6 (a). The TEM pictures are used to estimate size and shape, as well as verify that the processed samples are nanocrystalline. Figure 6 (b and c) displays the area and particle size distribution, showing that the majority of the particles are between 6 and 11 nm in size. The discrepancy between the TEM and XRD grain sizes is attributed to the different measurement principles: XRD provides the average crystallite size based on coherent diffraction domains, which may include multiple smaller grains or sub-domains, while TEM measures the physical particle size, which may include amorphous or polycrystalline regions.

### Fourier transform infrared spectroscopy (FT-IR) studies

**Fig. 7:**
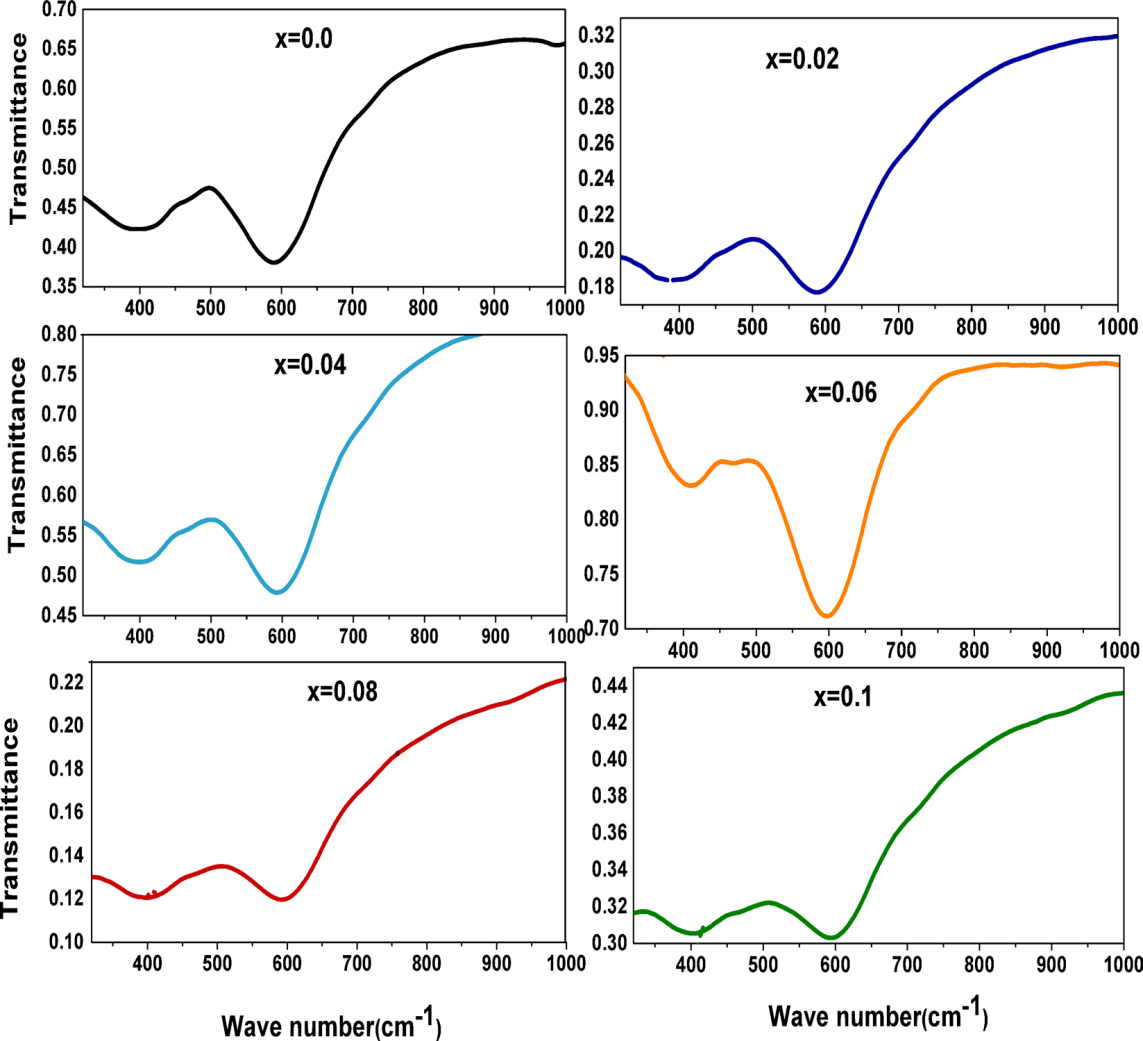
FT-IR spectra of as-prepared spinel ferrite **Co**_**1−2x**_**Ag**_**x**_**Cr**_**x**_**Fe**_**2**_**O**_**4**_ samples.

The spectrum of Co_1−2x_Ag_x_Cr_x_Fe_2_O_4_ samples is shown in Fig. 7, and the creation of the spinel ferrite structure is revealed by the two distinct absorption bands in this spectrum. The metal-oxygen bond’s tetrahedral lattice vibration is responsible for the high-frequency band υ_1_ at about 600 cm^−1^, whereas the metal-oxygen bond’s octahedral sublattice vibrations are responsible for the band υ_2_ at around 400 cm^−1^. There are shifts in the positions of υ_1_ and υ_2_ to high frequency with increasing Cr content *x* except at *x* = 0.02 and 0.08 may be due to Ag (As shown in Table 4), this behavior may be according to the variation of bond length of tetrahedral and octahedral sites (As shown in Table 4) which results from the estimation of cation distribution from XRD data^43^.

The following Eq. (13) is used to get the force constants at the tetrahedral site (F_1_) and octahedral site (F_2_) of the ions^44^.

12 $$\:F=4{\pi\:}^{2}{c}^{2}{{\upupsilon\:}}^{2}m$$

where *m* is the reduced mass of the Fe^3+^ and O^2−^ ions, which is approximately equal to 2.601 × 10^−23^ g, $\:{\upupsilon\:}$ is the tetrahedral or octahedral sites vibration frequency, and C is the velocity of light.

The following Eqs. (13, 14, 15, 16) using the values of the lattice constant (*a*) are used to calculate the distances between magnetic ions in the tetrahedral and the octahedral sites which called the tetrahedral and the octahedral bond length (“L_A_ and L_B_”) respectively (as Table 4)^45^

13 $$\:{L}_{A}=\left(\frac{\sqrt{3}}{4}a\right)$$

14 $$\:{L}_{B}=\left(\frac{\sqrt{2}}{4}a\right)$$

The relationship between F_1_ and F_2_ concerning Cr and Ag content x is presented in Table 4. The bond lengths “L_A_” and “L_B_” between magnetic ions at sites A and B have been shown to shift with decreasing Co concentration and increasing concentrations of Cr and Ag ions. It is noted from Eqs. (14, 15) that L_A_ and L_B_ display the same behavior of lattice constant^45^ this may be due to the variation of the unit cell because of the doping of relatively larger Ag^+^(ionic radius ≈ 1.26Å) and small Cr^+3^ (ionic radius ≈ 0.62Å) ions instead of cobalt ions(ionic radiusCo^+3^ ≈ 0.68 Å, Co^2+^ (0.78 Å). Because of the shorter bond, the cations in the A-site often interact more strongly with the surrounding oxygen anions than do the cations in the B-site, which have a longer bond. Consequently, the tetrahedral site’s (F_1_) force factor is greater than the octahedral site’s (F_2_) force factor. By modifying the bond length, size, mass, and charge of the ions that create this link, the atoms’ vibrations are modified^46^.

**Table 4:** The absorption bands υ_1_ and υ_2,_ the force constants of tetrahedral and octahedral sites, and the bond length of tetrahedral and octahedral sites.

X	υ_1_ (cm^−1^)	υ_2_ (cm^−1^)	F_1_ (*N*/m)	F_2_ (*N*/m)	F_av_ (*N*/m)	L_A_ (Å)	L_B_ (Å)
0.00	590.2	395.93	254.7	114.62	184.66	3.617	2.954
0.02	588.27	389.61	253.04	110.99	182.01	3.619	2.955
0.04	594.05	397.32	258.03	115.43	186.73	3.618	2.954
0.06	595.98	408.89	259.71	122.25	190.98	3.619	2.955
0.08	592.2	393.46	256.43	113.20	184.81	3.62	2.956
0.10	592.13	399.25	256.37	116.55	186.46	3.621	2.957

### Elastic properties

XRD and IR spectral analysis are used to calculate the elastic characteristics of the as-prepared samples since their mechanical properties are important for incorporating nanomaterials into a functioning device. The elastic stiffness constants are calculated by Eqs. (15,16,17,18)^47–49^.

15 $$\:{C}_{11}=\frac{{F}_{av}}{a}$$

16 $$\:{C}_{12}=\sigma\:{C}_{11}/(1-\sigma\:)$$

17 $$\:{C}_{44}={V}_{s}^{2}\rho\:$$

18 $$\:{C}_{66}=({C}_{11}-{C}_{12})/2$$

where the elastic modulus tensor is represented as *C*_*11*_, *C*_*12*_, *and C*_*44*_^50^. The resistance to deformation in response to a shearing force applied across the (100) plane in the [010] direction is physically represented by the symbol *C*_*44*_. Likewise, the resistance to shear deformation caused by shear stress applied across the (110) plane in the [1$\:\stackrel{-}{1}$0] direction is represented by$\:\:\:\frac{({C}_{11}-{C}_{12})}{2}$, *F*_*a*v_ is the average force constant given in Table 3 and σ is the Poisson ratio that depends on porosity *P*, which is given from $\:\sigma\:=0.324\times\:\left(1-1.043P\right)$.

All calculated parameters are displayed in Table 4. Variation in *C*_*11*_ and *C*_*12*_ constants may be due to the tetrahedral and octahedral site cations distribution^51^. Every sample satisfies the criteria for conception requirement and is mechanically stable when (C_11_- C_12_) > 0, (C_11_ + 2 C_12_) > 0, and C_44_ > 0^52^.

The elastic compliances are noticeable in calculating the directional properties of elastic moduli; they are represented by the notations S_11_, S_12_, and S_44_, and they are created using the associated elastic constants C_11_, C_12_, and C_44_^53^. The results obtained using Eqs. (19,20,21) are also listed in Table 5

19 $$\:{S}_{11}=\frac{{C}_{11}+{C}_{12}}{\left[\left({C}_{11}-{C}_{12}\right).\left({C}_{11}+2{C}_{12}\right)\right]}$$

20 $$\:{S}_{12}=-\frac{{C}_{12}}{\left[\left({C}_{11}-{C}_{12}\right).\left({C}_{11}+2{C}_{12}\right)\right]}$$

21 $$\:{S}_{44}=1/{C}_{44}$$

**Table 5:** Calculated stiffness coefficient (C_11_, C_12_, C_44_ and C_66_), elastic compliances (S_11_ and S_12_) for **Co**_**1−2x**_**Ag**_**x**_**Cr**_**x**_**Fe**_**2**_**O**_**4**_ nanoparticle system.

X	Stiffness coefficients (GPa) C_11_C_12_C_44_C_66_				Elastic compliances (10^−11^) Pa^−1^ S_11_ S_12_	
0.0	218.07	14.78	72.69	101.65	0 0.463	−0. 29
0.02	214.89	17.83	71.63	98.53	0.471	−0. 36
0.04	220.91	18.51	73.64	101.20	0.459	−0. 36
0.06	225.50	17.12	75.17	104.19	0.448	−0. 32
0.08	218.24	22.34	72.75	97.95	0.467	−0. 43
0.1	219.78	20.17	73.26	99.80	0.462	−0. 39

The group of Eqs. (22,23,24) was used to calculate the variation of longitudinal elastic wave velocity V_L_, the transverse wave velocity V_S_, and the mean velocity V_m_^54^:.

22 $$\:{V}_{L}=\sqrt{\frac{{C}_{11}}{{D}_{XRD}}}$$

23 $$\:{V}_{s}=\frac{{V}_{L}}{\sqrt{3}}$$

24 $$\:{V}_{m}=\left[\right({V}_{s}^{3}+2{V}_{L}^{3})/(3{V}_{s}^{3}{V}_{L}^{3}\left)\right]^{-1/3}$$

Variations in the velocities may be due to the variation in the force constant and experimental density. The force constant varied with Cr and Ag content x. As reported earlier by Lakshmi et al.^55^. This behavior can be interpreted as the result of the difference in bond lengths between the tetrahedral and octahedral sites for the Cr concentration x, which is the magnetic distance between ions.

Since transverse waves take less energy to shake a particle perpendicular to the propagation of the wave than longitudinal waves do in the parallel direction, it is expected that V_S_ will be lower than V_L_^47^.

Other elastic moduli such as Young modulus (E), Rigidity modulus (R), and Bulk modulus (K) are calculated using stiffness constants as the following group of Eqs. (25,26,27)^56^

25 $$\:E=\frac{({C}_{11}-{C}_{12})({C}_{11}+2{C}_{12})}{{C}_{11}+{C}_{12}}$$

26 $$\:K=\frac{1}{3({C}_{11}+{C}_{12})}$$

27 $$\:R=\frac{E}{2(\sigma\:+1)}$$

All measured values of the elastic parameters of the as-prepared Co_1−2x_Ag_x_Cr_x_Fe_2_O_4_ nanoparticle system are reported in Table 6. The observed Variation in G, K, and E may be attributed to the strengthening of the interatomic binding of CoFe_2_O_4_ and Ag, Cr doped CoFe_2_O_4_. Additionally, all samples’ Poisson’s ratio (σ) values were less than 0.1, which is consistent with the isotropic elasticity theory, which had values between 1 and 0.5^57^. To confirm the result, compare the theoretical results with the literature (as shown in Table 7) ^58,59^.

**Table 6:** Calculated elastic moduli (G, K, and E) in GPa and position, s ratio σ, longitudinal velocity VL, transverse velocity Vs, and mean velocity Vm as prepared **Co**_**1−2x**_**Ag**_**x**_**Cr**_**x**_**Fe**_**2**_**O**_**4**_ nanoparticle system.

X	G _(Gpa)_	K _(Gpa)_	$\:\varvec{\sigma\:}$ _(unitless_)	E _(Gpa)_	V_L_ _(m/s)_	V_s_ _(m/s)_	V_m_ _(m/s)_
0.0	101.65	82.54	0.0635	216.197	1803.58	1041.30	1156.03
0.02	98.53	83.52	0.0766	212.161	1741.43	1005.41	1116.20
0.04	101.20	85.98	0.0773	218.05	1805.08	1042.16	1157
0.06	104.19	86.58	0.0706	223.083	1889.59	1090.96	1211.17
0.08	97.95	87.64	0.0929	214.087	1756.34	1014.02	1125.76
0.1	99.80	86.71	0.08407	216.384	1788.42	1032.54	1146.32

**Table 7:** Comparison between our study and the literature.

Study	Method	Young’s Modulus (E, GPa)	Bulk Modulus (K, GPa)	Shear Modulus (G, GPa)
This work (x = 0.0–0.1)	IR-derived	212–223	82–88	98–104
Tamboli et al. (2025)	Ultrasonic	195–210	75–85	90–100
Ansari et al. (2025)	Nanoindentation	200–220	80–90	95–105

### Vibrating sample magnetometer (VSM) studies

**Fig. 8:**
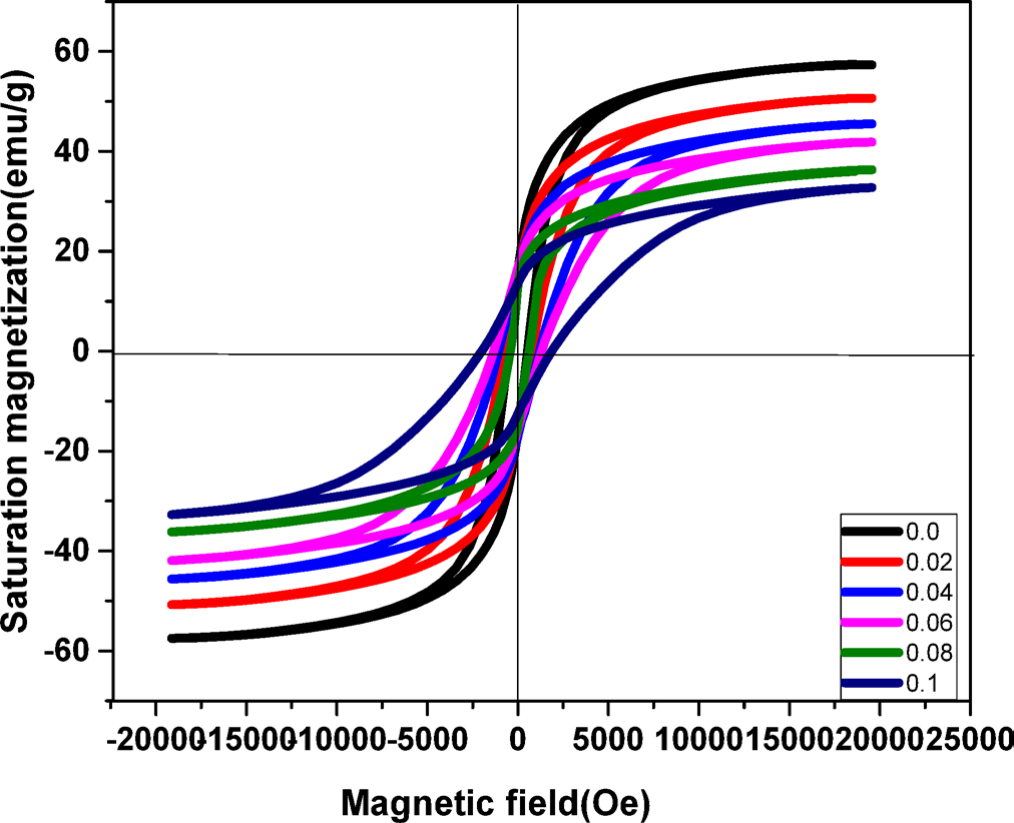
hysteresis loops of as-prepared **Co**_**1−2x**_**Ag**_**x**_**Cr**_**x**_**Fe**_**2**_**O**_**4**_ (x = 0, 0.05, 0.1, 0.2, 0.5) spinel ferrite samples.

**Fig. 9:**
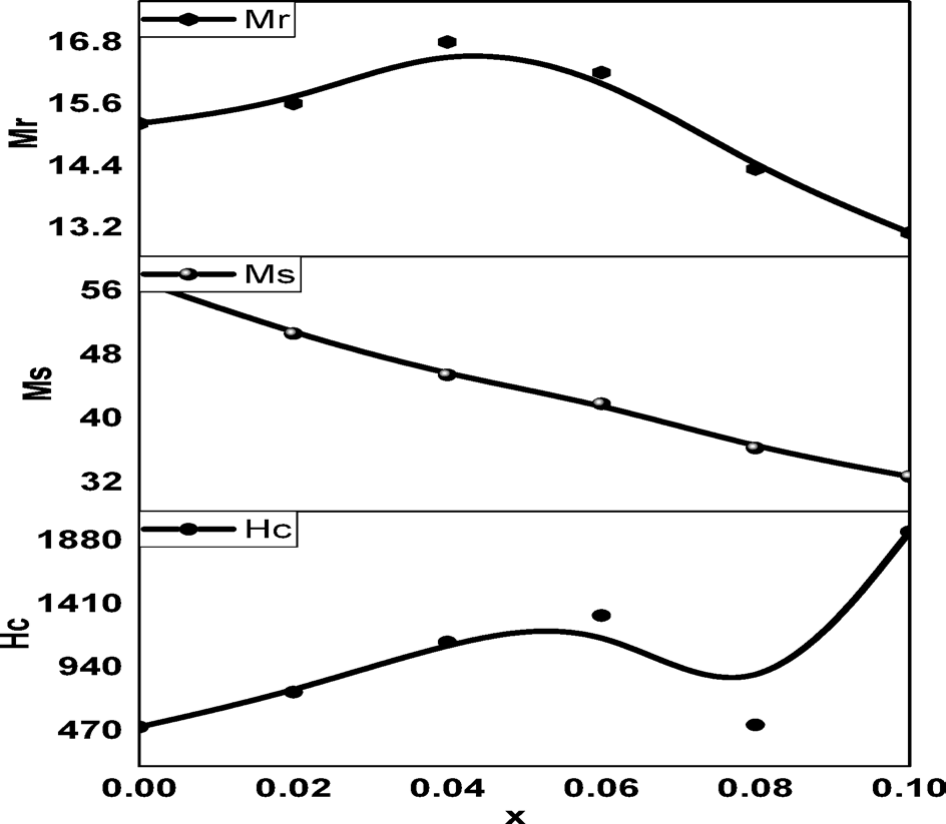
Variation of the saturation magnetization, coercivity, and remnant magnetization with x in as-prepared Co_1−2x_Ag_x_Cr_x_ Fe_2_O_4_ (x = 0, 0.05, 0.1, 0.2, 0.5) spinel ferrite samples.

**Fig. 10:**
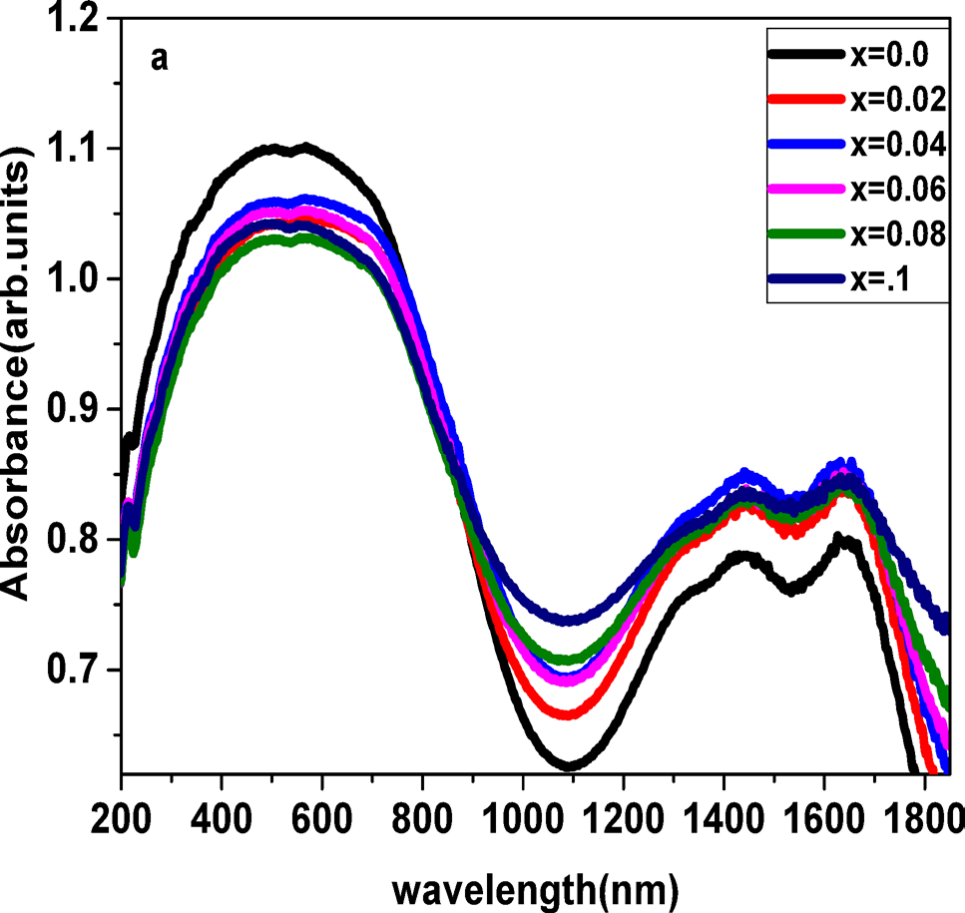
Variation of absorption as a function of wavelength.

A vibrating sample magnetometer (VSM) depicted in Fig. 8 and 9 was used to record magnetic hysteresis loops of pure CoFe_2_O_4_ at ambient temperature and the Cr, Ag-doped CoFe_2_O_4_ nanoparticles. The samples exhibit hard magnetic material behavior based on these hysteresis loops. Table 8 presents the magnetic properties that were obtained from the M-H graph. These include the Yaffet-Kittel angles $\:{\theta\:\:}_{Y-K}$, the theoretical magnetic moment m_th_, the experimental magnetic moment m_exp_, the coercivity H_C_, the saturation magnetization M_S_, and the residual magnetization M_r_. Figure 10 illustrates the reverse connection between coercivity H_C_ and saturation magnetization M_S_, where coercivity H_C_ and squareness ratio S decrease as x increases, but saturation magnetization M_S_ and remnant magnetization M_r_ increase. Octahedral sites M_B_ and tetrahedral sites M_A_ are two significant elements that affect magnetism in ferrites. Equation (28) provides the total magnetic moment M_th_.

28 $$\:\text{M}\text{t}\text{h}\:=\:|{\text{M}}_{\text{B}}-{\text{M}}_{\text{A}}|$$

According to Neel’s two-sublattice model and the core-shell model of ferrimagnetism, the magnetic moments of the ions in the spinel ferrites are arranged in a collinear structure, with two accessible sites. The increase in coercivity (Hc) at intermediate doping levels can be explained by the core-shell model. Doping introduces surface disorder and strain, enhancing surface anisotropy and pinning of domain walls, which increases Hc. At higher doping levels, however, excessive disorder may reduce anisotropy, leading to a drop in Hc. The Bohr magneton’s magnetic moment $\:{\text{M}}_{exp}\:$was calculated using Eq. (29)^60^.

29 $$\:{\text{M}}_{exp}=\frac{{M}_{w}x{M}_{s}}{5585}$$

where$\:\:{\text{M}}_{exp}$ is the magnetic moment in µ_B_, $\:{M}_{w}$ is the sample molecular weight and $\:{M}_{s}$ is the saturation magnetization, and 5585 is the magnetic factor^61^. Table 6 shows similar behavior for both M_exp_ and M_th_. A potential cause for the significant decrease in M_exp_ values from the M_th_ values is the Yaffet and Kittel (Y–K) model, which results from the nonlinear spin distribution in the system. A calculation of the spin Y–K angle (θ_Y−K_) has been made using Eq. (30)^60^ Table 7.

30 $$\:{M}_{exp}={M}_{B}{cos\theta\:}_{y-k}-{M}_{A}$$

However, by considering the relationship between the H_C,_ M_S,_ and K, which may be found in the Eq. (31)^62^

31 $$\:K=\frac{{H}_{c}x{M}_{s}}{0.96}$$

Spinel ferrites can interact in three different ways: (A)-(A), [B]-[B], and (A)-[B]. The greatest interaction is the (A)-[B] super-interaction^63^. From Table 8, the saturation magnetization and the magnetic moment decrease with increasing Silver and Chromium substitution. The first reason is that the chromium ions preferentially occupy octahedral sites, and the Fe^3+^ ions migrate to the tetrahedral sites, and the magnetic moments of Cr order anti-ferromagnetically with Fe ions’ magnetic moments, so increasing Cr content decreases the net magnetic moment of the B-site^64^ of B-site resulting in the decrease of $\:{M}_{s}$ 65. The second reason that substantially decreases the magnetization of CoFe_2_O_4_ is the substitution process in the spinel structure by the diamagnetic Ag. The B-site ionic radii can explain the magnetic result, and the ionic magnetic moment of Cr^3+^(3µ_B_) and Fe^3+^(5µ_B_) may have a greater non-collinear-shell-region than that of the sample’s smaller ferrite core crystallite size^65,66^. It is also possible that the growing number of uncoordinated magnetic spins on the shell layer surface that cannot align with the direction of the external magnetic field is the reason for the reduction in saturation magnetization of Ag, Cr doped CoFe_2_O_4_^67^.

**Table 8:** The experimental magnetic parameters: Ms (emu/g), Hc (Oe), and Mr (emu/g).

Cr and Ag content(x)	Ms(emu/g)	$\:\varvec{H}\varvec{c}\left(\varvec{O}\varvec{e}\right)$	M_*r*_(emu/g)
0.0	57.3	500	15.23
0.02	50.7	758	15.62
0.04	45.5	1131	16.81
0.06	41.9	1327	16.22
0.08	36.3	515	14.35
0.1	32.8	1947	13.11

The size of the nanoparticle Co_1−2x_Ag_x_Cr_x_Fe_2_O_4_ is obtained from BET measurement and found to range between 9.3–13 nm. If the magnetic diameter D_m_ in nanoparticles is tiny, the thermal energy surpasses the anisotropy energy, resulting in random ion movement and no strong preferred orientation for magnetization. The paramagnetic property (super-paramagnetic) of the grains of prepared Co_1−2x_Ag_x_Cr_x_Fe_2_O_4_ nano-ferrites is a result of the above. By measuring the magnetization loop’s slope at near-zero magnetic fields, the magnetic diameter D_m_ can be found. Superparamagnetic effects are known to be more prominent in larger particles^68^. The Eq. (37) was used to determine the magnetic diameter D_m_’s upper limit, and the results were listed in Table 9: (32).

32 $$D_m = \left[\frac{18k_{B}(dM/dH)_H \to \:0}{\pi \: \rho \:M_s^2}\right]^ \frac{1}{3}$$

where ρ is the density of the samples, *k*_*B*_ is the Boltzmann constant [69], and $(dMd/H )_{H\to\:0}$ is the initial slope near the origin is calculated by curve-fitting to the linear portion of the data from the hysteresis loop.

**Table 9:** The experimental magnetic parameters: saturation magnetization (M_S_), coercivity (H_C_), and remanent magnetization (M_r_), and the calculated magnetic parameters estimated from hysteresis loops of Co_1−2_xAg_x_Cr_x_Fe_2_O_4_ system the squareness ratio (S = $\:{M}_{r}$/$\:{M}_{s}$).

Cr and Ag content	Ms (emu/g)	$\:\varvec{H}\varvec{c}$ $\:\left(\varvec{O}\varvec{e}\right)$	M_*r*_ (emu/g)	S_Q_=M_*r*_/M_s_	M_the_	K (erg/oe)	M_exp_	Θ_y−k_ (degree)	D_nm_
0.0	57.3	500	15.23	0.26	4.38	29,883	2.736	37.68	8.4
0.02	50.7	758	15.62	0.3	5.87	40,052	2.136	37.26	7.7
0.04	45.5	1131	16.814	0.36	5.75	53,611	1.925	38.15	7.4
0.06	41.9	1327	16.22	0.387	5.57	57,890	1.778	38.07	7.7
0.08	36.3	515	14.35	0.397	5.25	19,516	1.782	39.12	10.7
0.1	32.8	1947	13.11	0.40	5.1	66,485	1.401	40.26	7.3

### UV- VIS studies

The simplest method to determine the band structure of semiconducting and non-metallic materials is optical absorption^69^. Figure 10 displays the absorption spectra of the produced materials that were recorded using UV-VIS spectroscopy in the range of wavelengths between 200 and 1800 nm. A wavelength that was almost equivalent to 523 nm corresponds to d–d transitions of Fe³⁺ ions in octahedral coordination and possibly charge transfer transitions between Fe²⁺ and Fe³⁺. Additionally, oxygen vacancies or Ag-related defect states may contribute to sub-bandgap absorption. All of the examined samples showed increased absorption. This might be a result of the material absorbing the residual photon energy after the incoming photon beam has enough energy to move electrons across the valence band into the conduction band. This may be because the incident photon beam lacks the energy to interact with atoms and passes through the material without providing any absorbance, which results in a rise in transmittance. After that, transmission increases with increasing wavelength, reaching a maximum of 1100 nm^70^.

The equation of the absorption coefficient (α) is used to determine the energy band gap (E_g_) with the Tauc plot Eq. (33). The equation can be as:

33 $$\:\left(\text{h}\text{v}\right)\text{n}=\text{k}(\text{h}\text{v}-\text{E}\text{g})$$

**Fig. 11:**
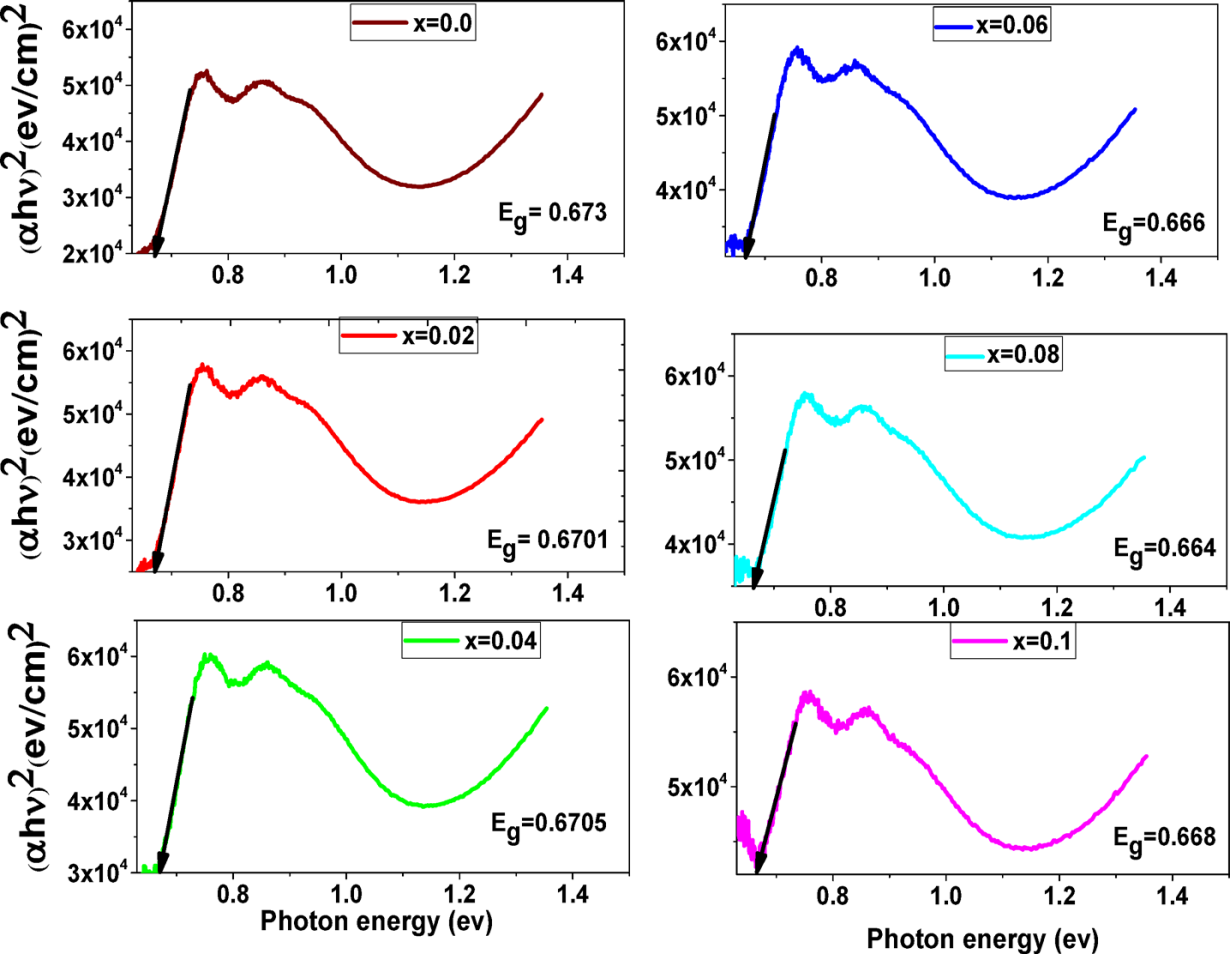
Variation of (αhʋ)^2^ with photon energy.

**Fig. 12:**
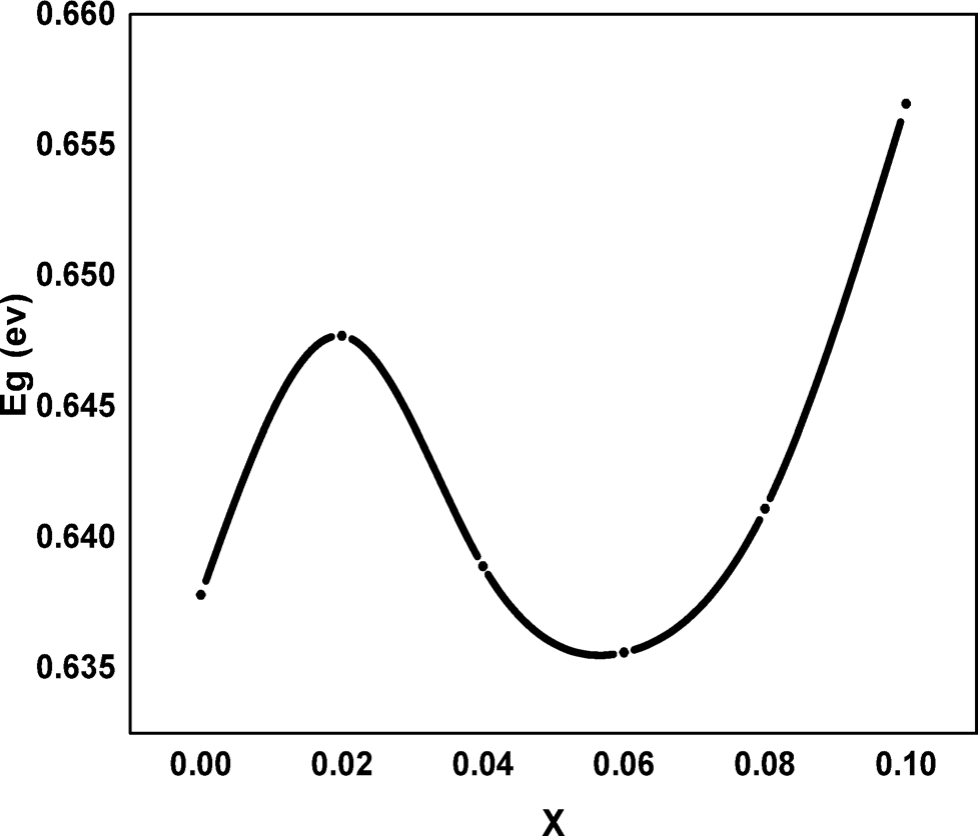
Variation of band gap energy with x.

Where α-absorption coefficient (α = 2.303 A/L), L is the thickness, A is absorbance (i.e. 1 cm in any case), h-planks constant (6.62607*10^−34^m^2^kg/s), ʋ-frequency (Hz), E energy independent constant, E_g_-optical band gap (eV), nature of transmission equal 2 for direct band gap material or ½ for indirect band gap material. To find the optical band gap energy, a graph was made between (αhʋ)^2^ and photon energy (hʋ). Figure 11 shows the extrapolation of the linear portion of the curve to (αhʋ)^2^=0 (for x = 0.0, 0.02, 0.04, 0.06, 0.08, and 0.1). The variation of E_g_ with Ag, Cr doped CoFe_2_O_4_ shown in Fig. 12 may be attributed to chemical defects, the average change in grain size, and structural disorder. For all the prepared samples, the values of E_g_ show semiconducting behavior, also the small values of E_g_ make these samples a potential candidate for photocatalytic applications.

### Impedance analysis

**Fig. 13:**
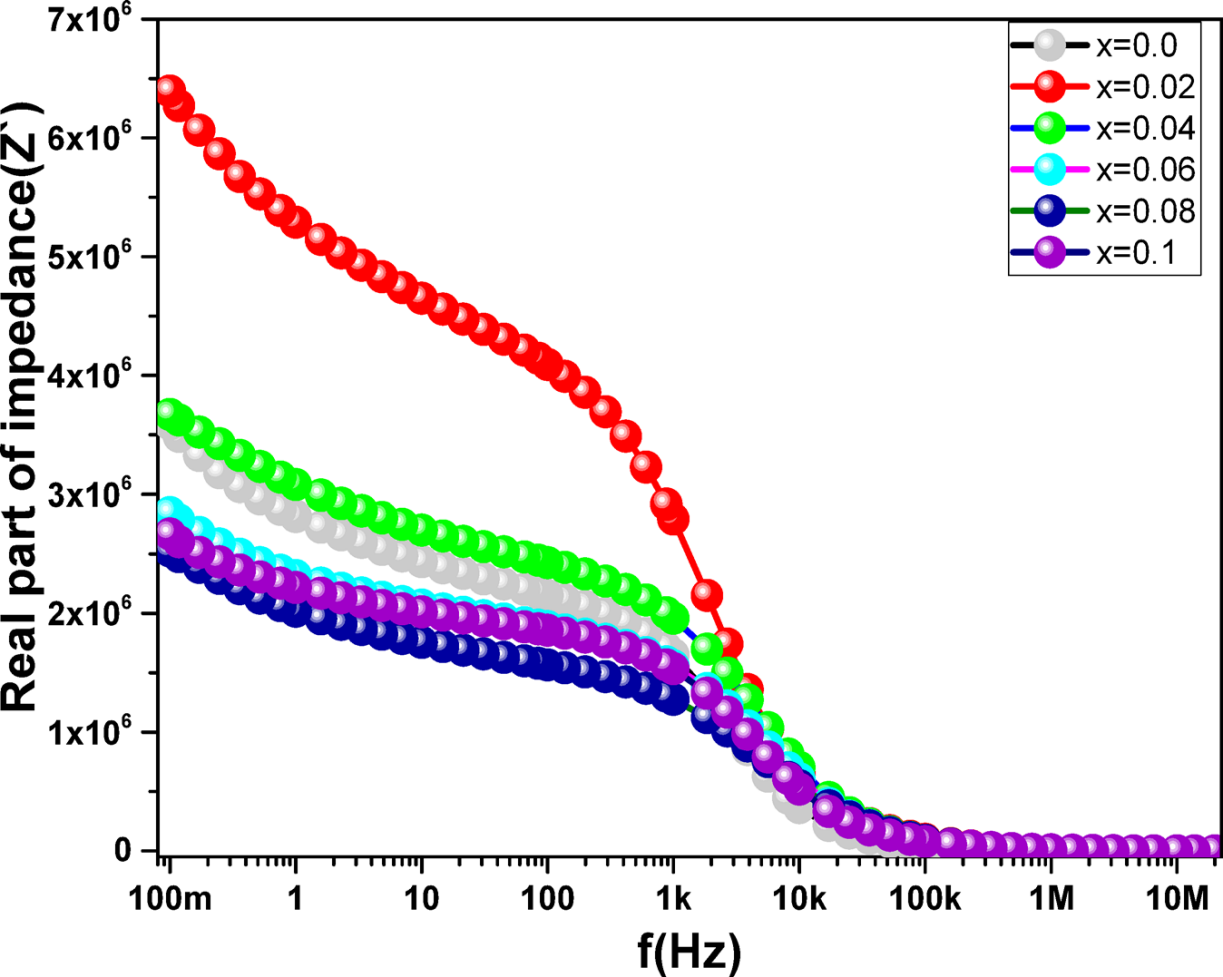
The frequency dependence of (a) $\:{z}^{{\prime\:}}$ for the **Co**_**1−2x**_**Ag**_**x**_**Cr**_**x**_ **Fe**_**2**_**O**_**4**_ samples.

**Fig. 14:**
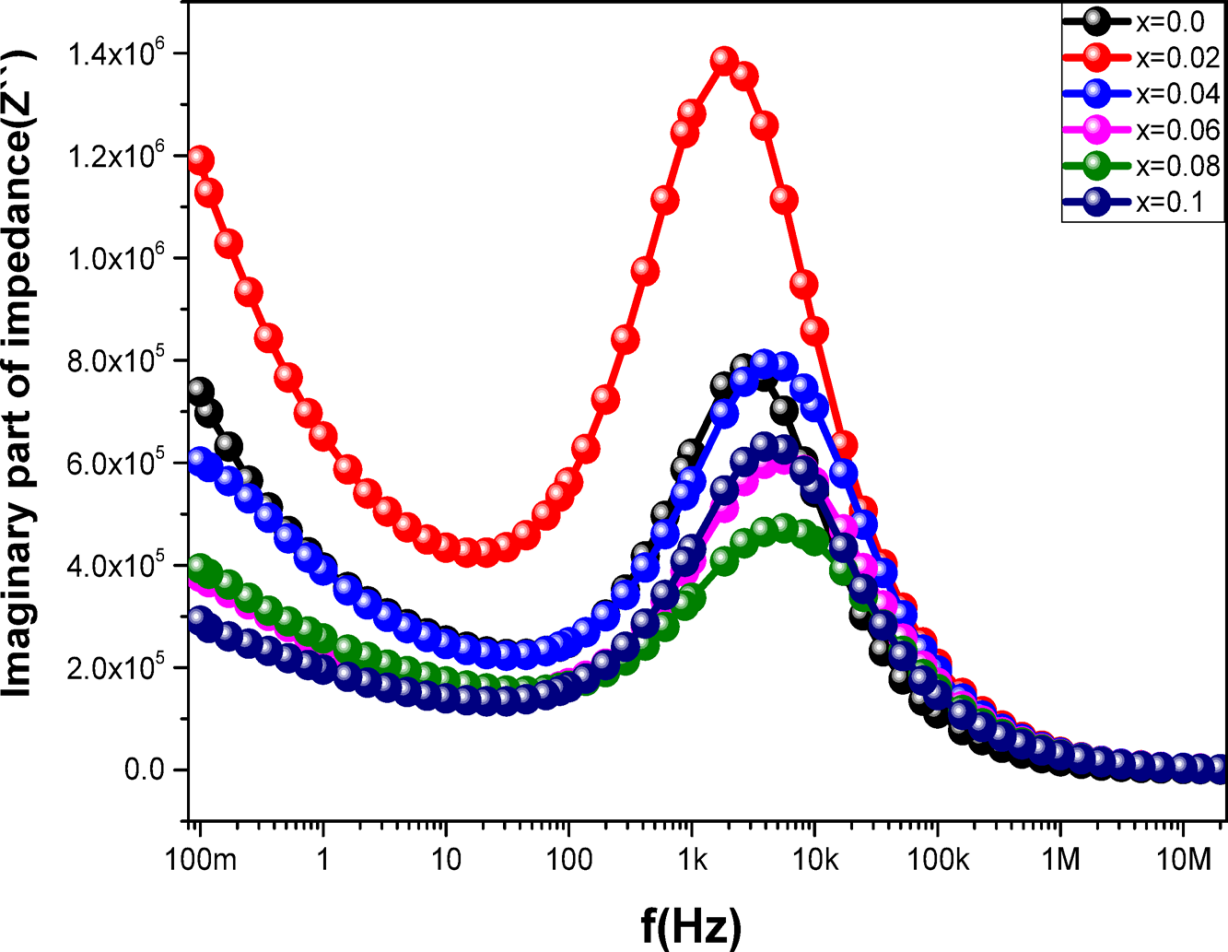
The frequency dependence of (b) $\:z"$ for the **Co**_**1−2x**_**Ag**_**x**_**Cr**_**x**_**Fe**_**2**_**O**_**4**_ samples.

Figures 13 and 14 show the variation of the ferrite samples’ impedance, which includes the real portion (Z′) and imaginary component (Z″) spectra depending on frequency. The field energy that is reversibly stored in the system is attributed to Z′ and Z′′. The impedance, represented by the imaginary Z″, displays the non-dissipative part of the response, which is regarded as an ideal capacitor through charging or an ideal, zero resistance inductor, where energy is converted into magnetic field energy around the inductor (the reactive part of impedance). The dissipative portion of the response, or the energy lost to the thermal reservoir from the input field (the resistive part of impedance), is shown by the real Z′. Over the frequency range of 10 − 1 to 107 Hz, the samples’ Z′ and Z” spectra show three different sectors, as shown in Figs. 13 and 14. Z′ and Z” values in the first sector drop as the frequency is increased up to 10 Hz. The second sector has frequencies ranging from 10 Hz to 100 kHz. The competition between the grain and grain boundary responses is constant as the frequency rises, and the Z′ and Z” behavior perfectly demonstrates the conversion of the resistive response into a capacitive response. Frequencies above 100 kHz are represented by the third sector. Z′ and Z” values exhibit constancy as the frequency rises, however Z″ values still show a little decline as the frequency rises, reaching very low values.

**Fig. 15:**
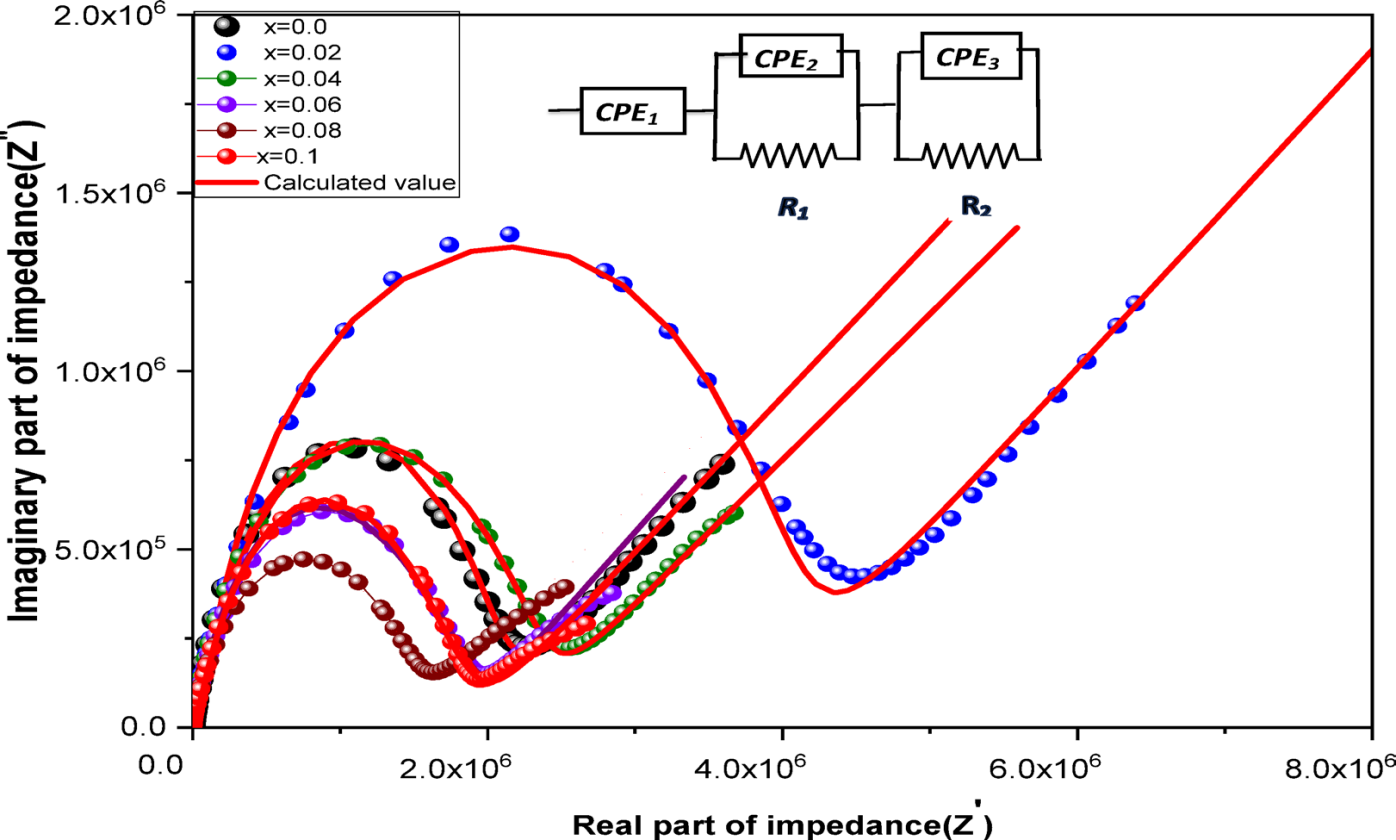
, The Nyquist plots for **Co**_**1−2x**_**Ag**_**x**_**Cr**_**x**_ **Fe**_**2**_**O**_**4**_ and the electrical model used for the fitting of the Nyquist diagram.

An equivalent circuit is used to illustrate the typical configuration produced by the Z′/Z″ Nyquist diagram^71,72^, which is correlated with the physical processes taking place in the system being studied. Figure 15. The low-frequency region’s Z′ vs. Z″ curves demonstrated a fast linear decline with increasing frequency, indicating improved access for free charges to the electrode and inter-granular electrical resistance that supports the segment dipoles that cause electrode capacitance. The semicircular arcs of the col-col plot enhance the examination of grain and grain boundary effects. When the grain boundary resistance was greater than the bulk resistance, a semicircular arc formed. Grain boundaries are explained by the first semicircle arc shape, whereas the effects of both the grain and the electrode are explained by the second semicircle arc shape^73^. Variations in the semicircle diameters reveal variations in the samples’ grain boundaries and internal resistance.

CPE_1_ is the equivalent series capacitance (interface capacitance between the electrode and the sample’s first side (1st)); R_1_/CPE_2_ and R_2_/CPE_3_: are resistance and capacitance of the charge transfer through grain boundary and charge transport through grain; resectivelly^74^. n is a real number between 1 and 0, the more ideal the capacitive behavior of the CPE when n gets closer to 1. R and CPE values are presented in Table 10. These results proved the effect of Ag-Cr ions content giving optimization of both the resistive and capacitive behavior for the sample x = 0.02. As Ag-Cr concentration has increased, this impact has diminished, leading to many variances in R and CPE values at a given frequency. The electrostatic interactions between the Cr content in the octahedral sites and the Ag concentration in the spinel matrix surfactant, in addition to the charge transfer and charge density within the nanograin spinel crystal and the external alternating electric field, respectively, determine the magnitude of each quantity under numerous conditions^75^.

**Table 10:** Variation of impedance parameters at room temperature.

X	*R*_1_(Ω)	*R*_2_(Ω)	CPE_1_	*n*_1_	CPE_2_	*n*_2_	CPE_3_	*n*_3_
0.0	9 × 10^5^	9.8 × 10^5^	3 × 10^−7^	0.26	4.99 × 10^−11^	0.83	1.99 × 10^−11^	0.89
0.02	1.6 × 10^6^	2.2 × 10^6^	1.4 × 10^−7^	0.27	3.06 × 10^−12^	0.87	1.21 × 10^−11^	0.84
0.04	8.7 × 10^5^	1.3 × 10^6^	3.02 × 10^−7^	0.25	8.88 × 10^−11^	0.76	7.09 × 10^−12^	0.85
0.06	9.6 × 10^5^	8.7 × 10^5^	6.8 × 10^−7^	0.28	6.60 × 10^−10^	0.66	1.54 × 10^−11^	0.899
0.08	7.4 × 10^5^	6.8 × 10^5^	5.1 × 10^−7^	0.24	5.45 × 10^−10^	0.66	1.21 × 10^−11^	0.88
0.1	6.1 × 10^5^	1.2 × 10^6^	1.03 × 10^−6^	0.25	6.01 × 10^−9^	0.54	3.08 × 10^−11^	0.87

The barrier height that charge carriers must cross to enter the lattice was evaluated to gain a fuller understanding of the **Co**_**1−2x**_**Ag**_**x**_**Cr**_**x**_**Fe**_**2**_**O**_**4**_ -transport characteristics. Based on the hopping model, the dielectric loss " may be characterized by Giuntini’s law^76^.

**Fig. 16:**
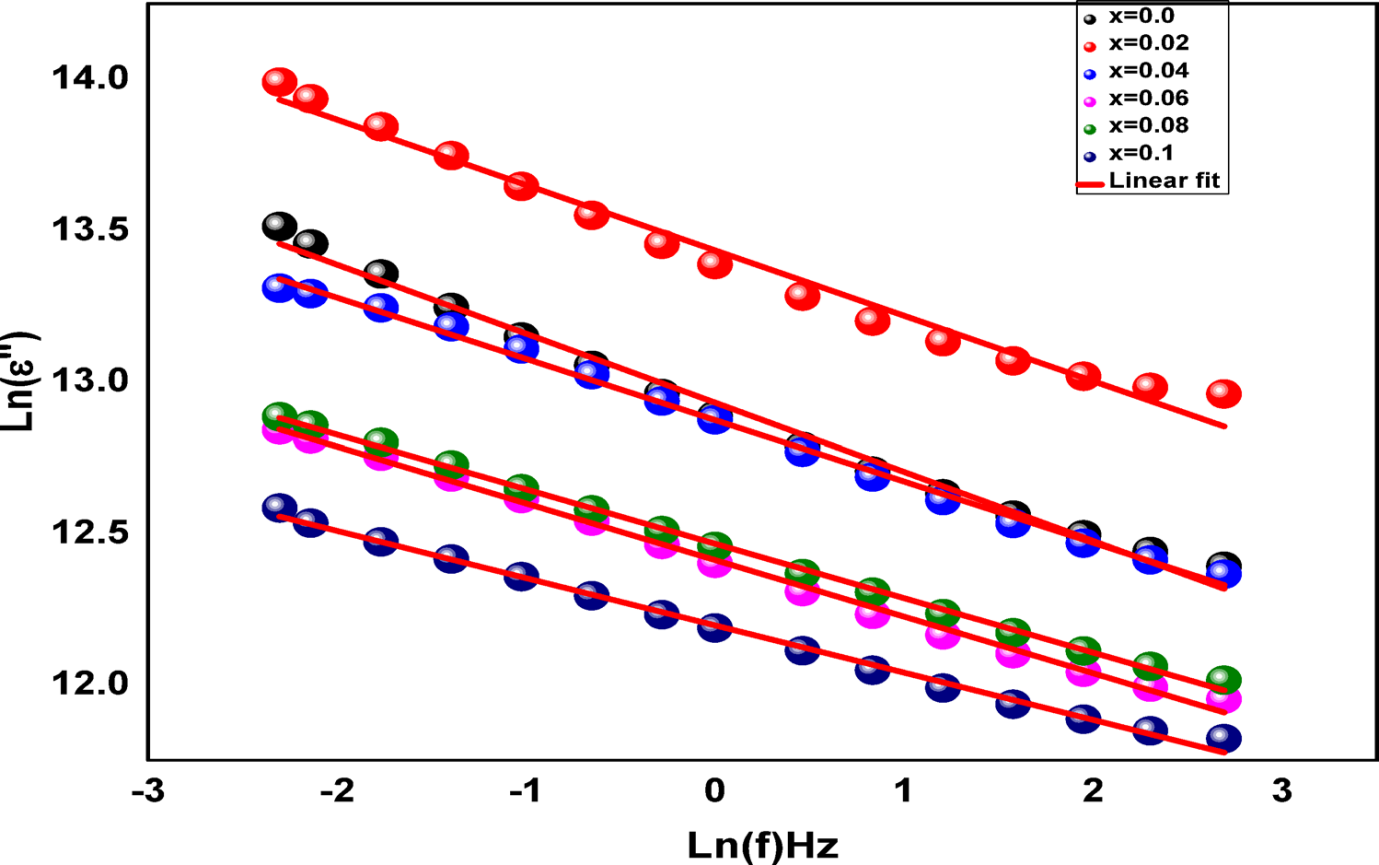
Variations in Ln(ε”) with frequency at different temperatures.

It can be summed up as follows:

34 $$\:\epsilon\:"\left(\omega\:\right)=A{\omega\:}^{m}$$

where A is a temperature-dependent constant, ω denotes the angular frequency, and m is an exponent indicating the strength of the interaction between the lattice’s dipoles. Concerning the maximum barrier height (W_m_), the latter is connected using the following expression^77^:

35 $$\:m=-\frac{4{K}_{B}T}{{W}_{m}}$$

where K_B_ is the Boltzmann constant and T is the temperature. Figure 16. shows the curve of Ln(ε”) versus Ln(ω). This can be used to obtain the m values, and Eq. (35) can then be used to estimate the maximum barrier height. The findings demonstrate that W_m_ rose from 0.45 to 0.66 eV with the increase of Cr and Ag concentration at room temperature. This makes sense since the charge carriers can converge at the grain boundaries to form barriers and the internal lattice dipoles can dissipate as the concentration of Ag and Cr increases. We have determined the minimum hopping distance R_min_ to carry out additional research into the electric characteristics of **Co**_**1−2x**_**Ag**_**x**_**Cr**_**x**_ **Fe**_**2**_**O**_**4**_. The binding energy values are used to compute it under Eq. (34).

36 $$\:{\text{R}}_{\text{m}\text{i}\text{n}}=\frac{2{\text{e}}^{2}}{{\uppi\:}{\epsilon}_{0}{\upepsilon\:}{\prime\:}{\text{W}}_{\text{m}}}$$

The R_min_ progression at room temperature is being measured at 100 Hz. The findings show that R_min_ drops from 220pm at x = 0.0 to 55 pm.at x = 0.1 as Ag and Cr concentration increase where e is the electron charge, $\:\epsilon\:{\prime\:}$ the dielectric constant and $\:{\in\:}_{0}$ the permittivity of free space. According to the information above, these samples could be used to make electronic devices in a range of devices whose electrical conductivities control their performance, like antennas and capacitors^78,79^.

### Dielectric studies

**Fig. 17:**
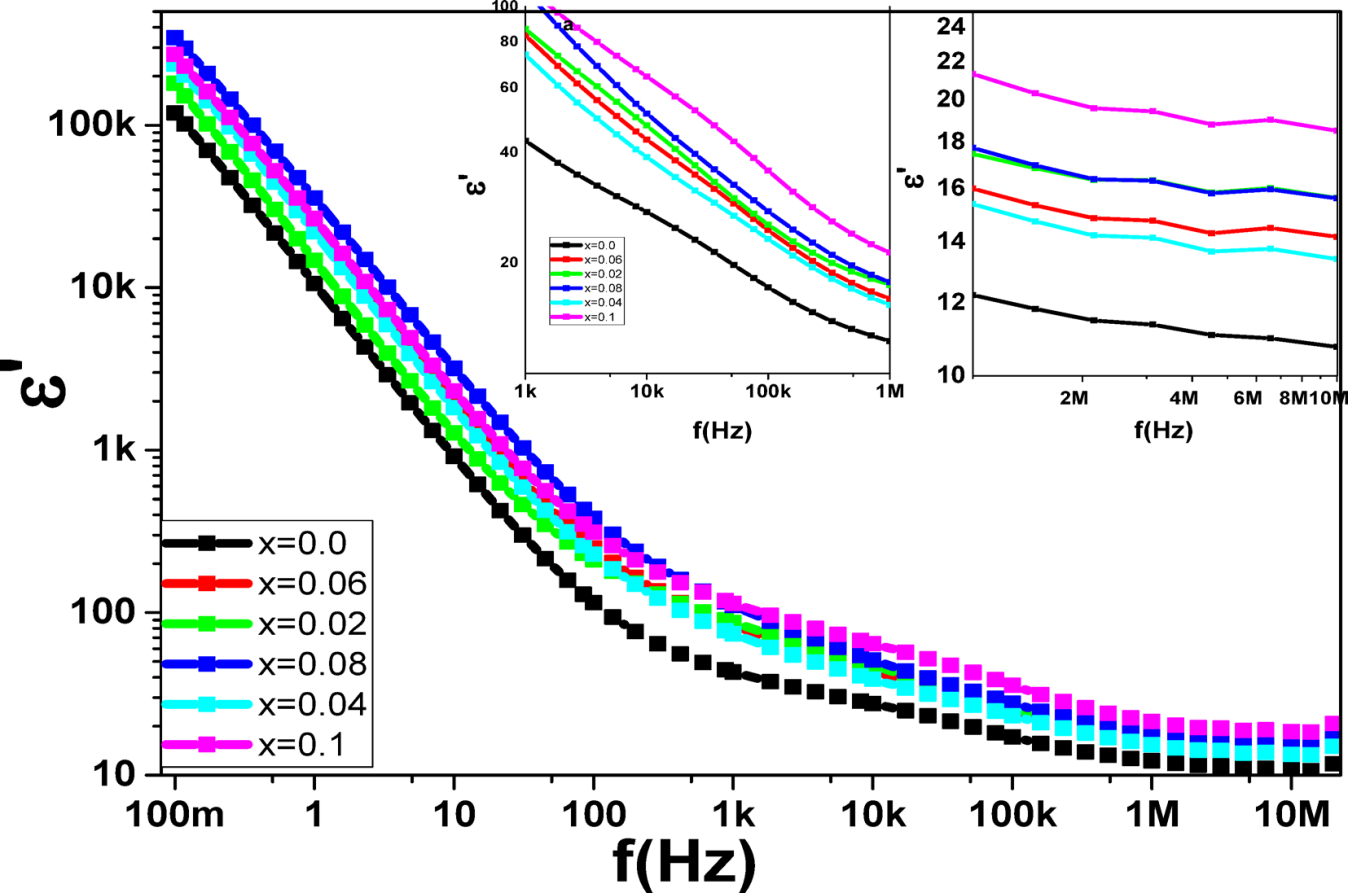
The frequency dependence of ὲ for the **Co**_**1−2x**_**Ag**_**x**_**Cr**_**x**_ **Fe**_**2**_**O**_**4**_ samples.

**Fig. 18:**
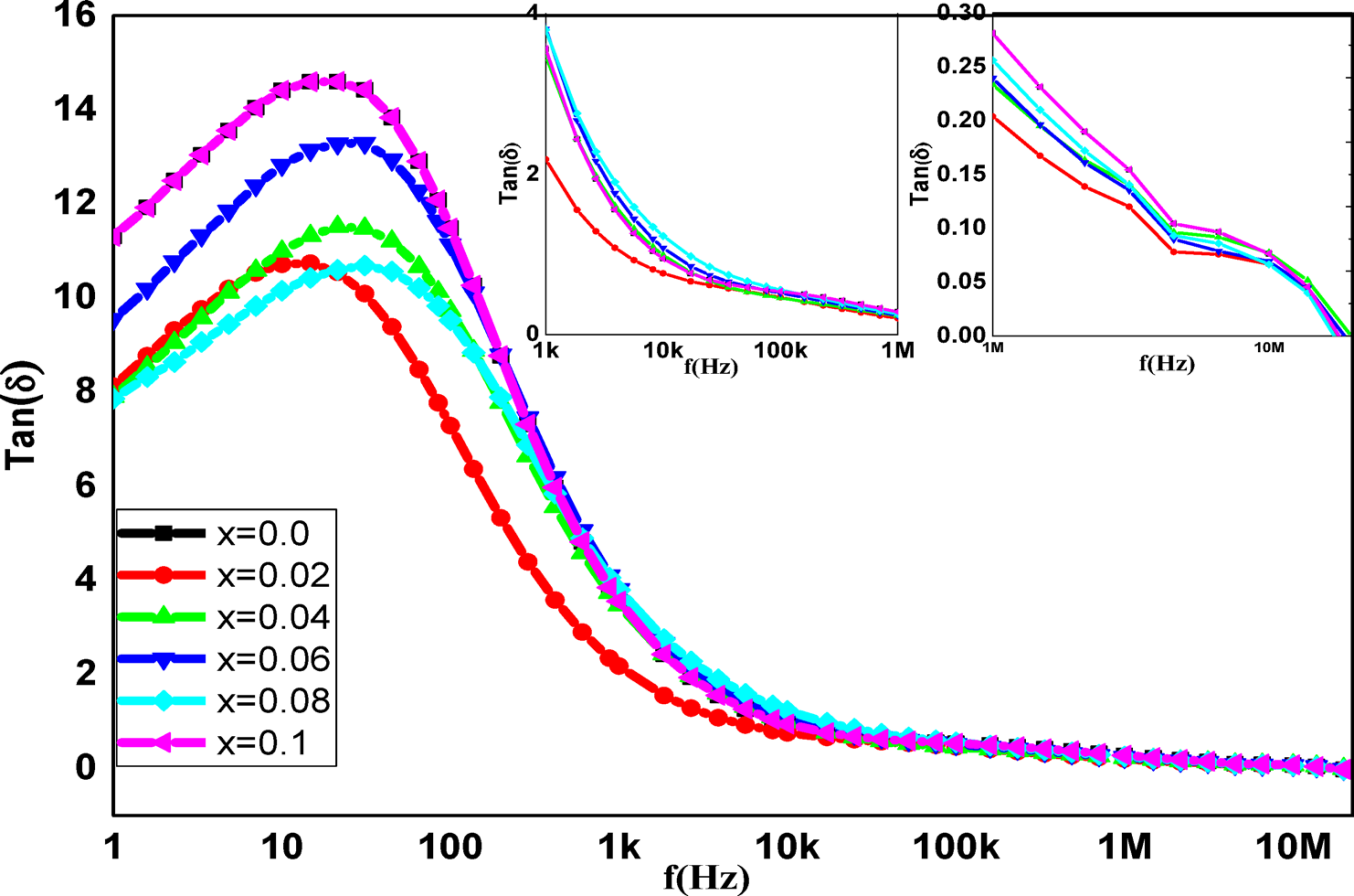
The frequency dependence of tan(δ) for the **Co**_**1−2x**_**Ag**_**x**_**Cr**_**x**_ **Fe**_**2**_**O**_**4**_ samples.

**Fig. 19:**
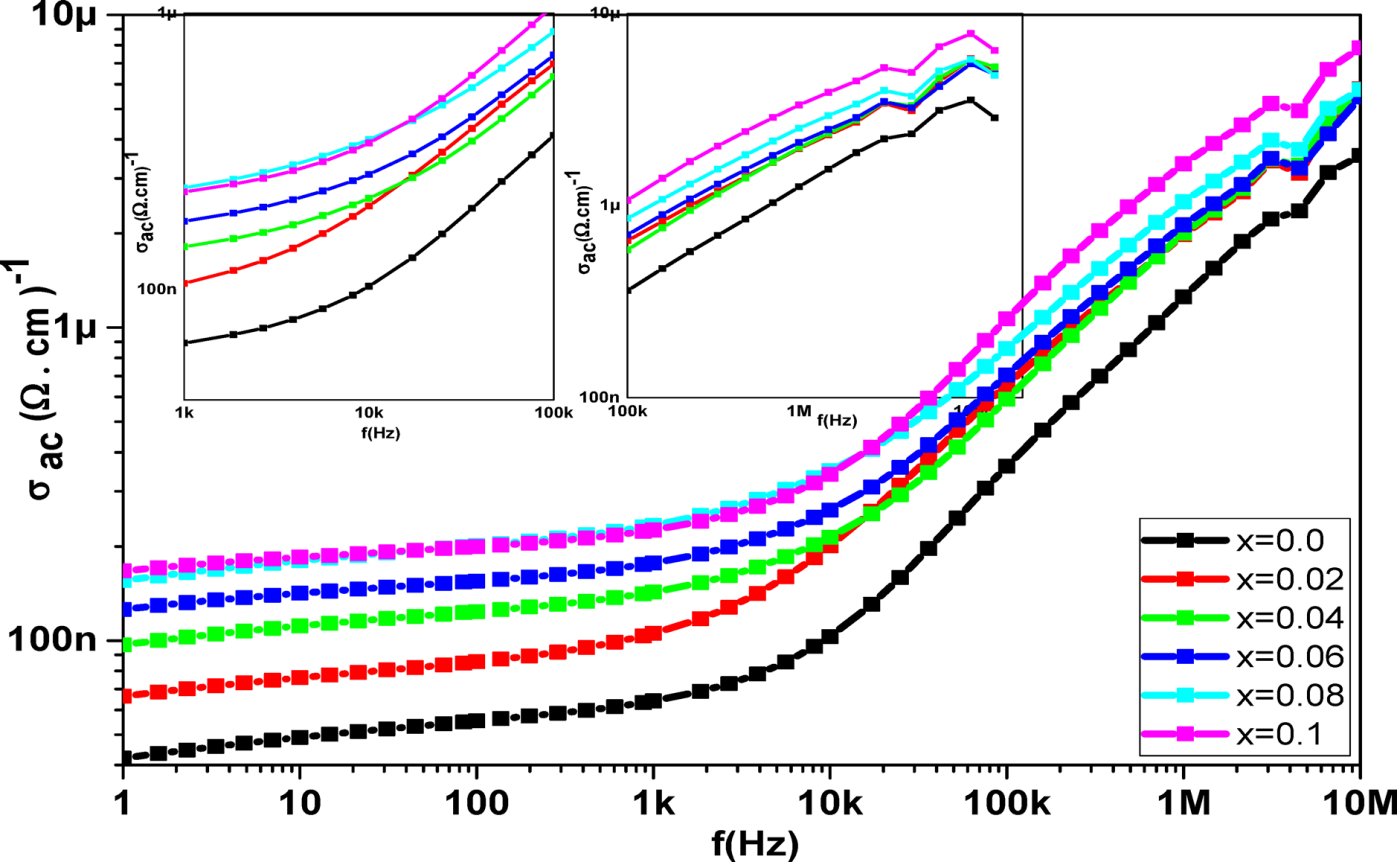
The frequency dependence of σ_ac_ for the **Co**_**1−2x**_**Ag**_**x**_**Cr**_**x**_ **Fe**_**2**_**O**_**4**_ samples.

Ferrite dielectric findings analysis provides insights into the characteristics of ferrite charge carriers and conduction mechanisms, such as the polarisation process. The dielectric behavior of magnetic materials was affected by temperature, grain size, synthesis and characterization techniques, frequency of applied field, cation distribution at A and B sites, and other factors^80^. Figures 17 and 18 show the dielectric measurements as a function of frequency (ὲ and tan(δ)), which show the same decreasing behavior with the frequency increasing, while the ac conductivity (σ_ac_) exhibits reverse behavior. The Maxwell-Wagner model, which explains the concept of grain and grain boundaries (thin insulating layers), supports Koops’ theory, which is the basis for how (σ_ac_, ὲ) and tan(δ) vary with the applied electric field frequency^81,82^. Variation of ac conductivity (σ_ac_) of **Co**_**1−2x**_**Ag**_**x**_**Cr**_**x**_ **Fe**_**2**_**O**_**4**_ samples with frequency shown in Fig. 19, the ac conductivity (σ_ac_) in the figure grows towards the high-frequency area, and as the frequency increases, the ac conductivity’s exponential expansion is accelerated^83^, Conversely, at lower frequency regions, the ac conductivity (σ_ac_) linearly increases in a frequency-dependent manner. This can be explained by the fact that in the lower frequency range, the grain boundaries act more strongly, giving the impression that the sample is highly resistant. Conversely, in the high-frequency range, the sample appears to have low resistance, suggesting that an increase in conductivity at higher frequencies may be the result of grain effects.

The ε′ and tan δ spectra of the samples under study exhibit three distinct zones within the frequency range of 10^−1^ to 10^7^ Hz, as illustrated in Figs. 17 and 18. ε′ and tan δ values in the first region sharply fall as the frequency is increased up to $\:{10}^{3}$ Hz. This behavior demonstrates that the electrode polarisation (EP) effect has a dominant contribution^84^. The tan δ spectra, on the other hand, are related to dipole segment polarisation and show the α-relaxation of the peak local electrode polarisation^85^. In the second region, starting from 1 kHz to 100 kHz, the non-linear values of ε′ decrease with the increasing frequency^49^, attributed to grain boundary polarization (interfacial polarization). The third region starts from the frequency above 100 kHz, while the tan δ values slightly decrease to reach very low values, the ε′ values approach a stable value with the frequency increasing, indicating that the high frequency limits the permittivity ε_∞_ values of the ferrite samples. The samples at these concentrations may be useful as a low-loss and low-permittivity dielectric substrate for the creation of radio frequency-dependent electronic devices^86^, as indicated by the significantly low tan δ values seen in the MHz range.

The addition of Ag-Cr ions causes Fe ions to move from the tetrahedral site into the octahedral site, which causes the dielectric permittivity values of the samples to trend upward. The accumulation of charge diffusion within grains, which results in dipole segments at electrodes and electrode polarisations, is the cause of the permittivity rise^85^. The increase of Cr and Ag ions concentration causes small variations of ε′ values at a given frequency, as seen in Fig. 17. At lower frequencies, both ε’ and the polarisation effects are high; however, as the frequency of the field increases, the values start to decrease. The reason for this is that the dipoles can follow the changed field at low frequencies, where they can do this during the change, but not at higher frequencies, when they cannot. Specifically, there is cause for concern at f = 12 Hz, where the value of tanδ increases. Higher frequencies cause the electric field to periodically reverse so fast that the larger dipole segments do not match the field’s orientation. This section starts by introducing the relaxation and ending the EP relaxation’s dominance. This broad peak rises against the general decrease with increasing frequency; these broad peaks may be attributed to dipole segments at electrodes [86,87].

Figure 19 shows that the σ’ values significantly improve with increasing frequency. The actual complex AC electrical conductivity component σ’ was determined at room temperature for a range of samples varying in Ag-Cr contents. When the frequency increases, the σ’ values also rise, exhibiting two distinct linear behaviors in two distinct regions on a logarithmic scale. The first one, from 0.1 Hz to 10k Hz, and the second, from 10k Hz to 10 MHz, show that low frequencies cannot cause any growth in σ’. This indicates that a threshold frequency is required to initiate the influence of frequency increases on rising σ’ values. The crystalline nature of the samples is indicated by the values of σ’ in the first section, which exhibit plateau behavior spanning the frequency range of 0.1 Hz to 10 kHz. According to XPS data, the hopping mechanism that allows electrons to hop between Fe^2+^↔ Fe^3+^ and holes to hop between Co^2+^↔Co^3+^ causes the values of σ’ in the second section to climb quickly as f increases^87^, where more and more mobile charge carriers are accumulated inside the grains. This will increase the charge carrier conductivity of the samples. This behavior of σ′ has been mentioned in many works earlier^46^.

In general, Jonscher’s power law applies to the frequency dependence of conductivity^88^

37 $$\:\sigma\:\left(\omega\:\right)={\sigma\:}_{dc}+A{\omega\:}^{n}$$

where A is a constant that controls the polarizability strength, $\:\sigma\:\left(\omega\:\right)$is the total conductivity, $\:{\sigma\:}_{dc}$ is the direct current conductivity of the sample, and $\:A{\omega\:}^{n}$ is the AC conductivity purely dispersive, where a power law characteristic in the angular frequency $\:\omega\:$ and exponent n (0 $\:\le\:$ n $\:\le\:$ 1)^89^ indicates the degree of interaction between mobile ions and the surrounding lattices. According to Jonscher, the origin of the conductivity’s frequency dependence can be connected to the relaxation phenomena of the ionic environment, which are caused by mobile charge carriers, This behavior arises due to the presence of mixed valence states (Fe²⁺/Fe³⁺ and Co²⁺/Co³⁺) and oxygen vacancies, which facilitate carrier mobility through extended hopping paths. As Ag and Cr ions are introduced, they induce lattice distortions and defect states that act as potential barriers for charge carriers. Although direct microstructural evidence is not available, the correlation between crystallite size reduction, porosity, and impedance behavior supports this interpretation. We have clarified this mechanism and suggested future studies using HRTEM or grain boundary mapping^90^. Plotting the sample’s experimental conductivity spectra is done using Eq. (37). An overview of the fitting results is shown in Table 11. For ionic conducting materials, the frequency exponent, or n, is frequently observed to range between 0.6 and 1^91^. The explanation for this is the mobile charge carriers’ hopping conduction via the barrier separating the two sites.

**Table 11:** The optimal fitting parameters derived from the conductivity ($\:\sigma\:$) experimental data as a function of frequency using jonscher’s power law.

X	$\:{\varvec{\sigma\:}}_{\varvec{d}\varvec{c}}$	A	*N*
0.00	2.2e-8	1.45e-9	0.48
0.02	4.9e-8	2.5e-9	0.48
0.04	7.8e-8	2.0e-9	0.49
0.06	9.5e-8	4.2e-9	0.44
0.08	11.5e-8	7.2e-9	0.415
0.10	9.4e-8	8.5e-9	0.42

Table 11 illustrates that all of the values of n are below one, suggesting that the hopping conduction process implied by charge transportation in the samples is thermally initiated inside the samples being studied. At room temperature, $\:{\sigma\:}_{ac}$ Values are found to be more than 100 times greater than $\:{\sigma\:}_{dc}$ Values. The behavior of the $\:{\sigma\:}_{dc}$ Values indicate how the Ag-Cr content affects the $\:{\sigma\:}_{dc}$ Values, which indicate structural alterations brought about by nonstoichiometric structure and uncompensated Ag ions between spinel structure sites. Because the electrostatic interactions across the functional groups of dipolar allow the individual dipoles of nano-ferrite grains to be distributed diffusively, a rise in σ_dc_ values suggests that these interactions are significant. This indicates that the non-compensated Ag ions and compensated Fe ions are evenly distributed throughout the tetrahedral and octahedral locations of the spinel matrix. This enhances the charge mobility since there are more conducting Ag ions in the spinel lattice environment. Consequently, the rate of charge migration increases, increasing the charge’s contribution to electrical conductivity. Additionally, Ag and Cr charge transfer complexes are incorporated, where charges are moved by electric fields and coulomb forces. This also unequivocally validates the existence of electrode polarization, or EP, which is caused by an accumulation of charge close to the electrode, which results in increased permittivity and decreased conductivity^92^. According to the following relation, the charge mobility ($\:{\mu\:}_{e}$), charge density, $\:{n}_{e}$, and the charge mobile value all influence the $\:{\sigma\:}_{DC}$ value^93^.

38 $$\:{\sigma\:}_{DC}={n}_{e}{\mu\:}_{e}$$

Thus, the increase in $\:{\sigma\:}_{DC}$ Values for the samples indicate that there is an increase in the charge mobility because of the excess of conducting Ag ions in the spinel lattice. Consequently, the rate of charge migration increases, increasing the charge’s contribution to electrical conductivity. Adding Ag and Cr charge transfer complexes allows charges to move via coulomb forces and an electric field^46,87^.

## Conclusion

XRD analysis of the **Co**_**1−2x**_**Ag**_**x**_**Cr**_**x**_**Fe**_**2**_**O**_**4**_ nanoparticles verified their single-phase cubic spinel structure. The saturation magnetization decreases as the Cr and Ag concentrations rise, which in turn decreases M_S_. The low Eg values of the produced samples imply that they may find usage in photocatalytic applications, and their E_g_ values all show a semiconducting nature. Although ac conductivity showed the opposite pattern, indicating possible applications, dielectric investigations showed that the dielectric constant decreased with frequency. These detailed understandings of the structural, magnetic, optical, dielectric, and electrical properties of **Co**_**1−2x**_**Ag**_**x**_**Cr**_**x**_**Fe**_**2**_**O**_**4**_ nanoparticles enable their potential application in technological domains such as sensors, capacitors, and other electronic devices, where their enhanced and customized properties will be advantageous.

Magnetic nanoparticles, particularly those with hard magnetic properties, are finding increasing applications in various fields, including biomedicine and data storage. In biomedicine, they are used for targeted drug delivery, imaging (MRI contrast agents), and cancer treatment (magnetic hyperthermia). In data storage, they offer the potential for high-density, stable storage solutions.

## Data Availability

All datasets presented in this study are included in the article and can be provided by the corresponding author upon reasonable request.
